# Targeting autophagy sensitises lung cancer cells to Src family kinase inhibitors

**DOI:** 10.18632/oncotarget.25213

**Published:** 2018-06-08

**Authors:** Ewa Rupniewska, Rajat Roy, Francesco A. Mauri, Xinxue Liu, Maciej Kaliszczak, Guido Bellezza, Lucio Cagini, Mattia Barbareschi, Stefano Ferrero, Anna M. Tommasi, Eric Aboagye, Michael J. Seckl, Olivier E. Pardo

**Affiliations:** ^1^ Division of Cancer, Department of Surgery and Cancer, Imperial College London, London, United Kingdom; ^2^ Department of Histopathology and Imperial College London, London, United Kingdom; ^3^ Statistical Advisory Service, Imperial College London, London, United Kingdom; ^4^ Institute of Pathology, Division of Cancer Research, Perugia Medical School, University of Perugia, Perugia, Italy; ^5^ Department of Thoracic Surgery, Division of Cancer Research, Perugia Medical School, University of Perugia, Perugia, Italy; ^6^ Unit of Surgical Pathology, Laboratory of Molecular Pathology S. Chiara Hospital, Trento, Italy; ^7^ Division of Pathology, Fondazione IRCCS Ca' Granda Ospedale Maggiore Policlinico, University of Milan, Milan, Italy

**Keywords:** lung cancer, SRC, dasatinib, resistance, apoptosis

## Abstract

Lung cancer is the main cancer killer in both men and women, mostly due to the rapid development of drug resistant metastatic disease. Here, we evaluate the potential involvement of SRC family kinases (SFK) in lung cancer biology and assess the possible benefits of their inhibition as a therapeutic approach. We demonstrated that various SRC family members, including LYN and LCK, normally expressed solely in hematopoietic cells and neural tissues, are overexpressed and activated in a panel of SCLC and NSCLC cell lines. This was clinically relevant as LYN and FYN are also overexpressed in lung cancer clinical specimens. Moreover, LYN overexpression correlated with decreased patient survival on univariate and multivariate analysis. Dasatinib (BMS-354825), a SRC/ABL inhibitor, effectively blocked SFK activation at nanomolar concentrations which correlated with a significant decrease in cell numbers of multiple lung cancer cell lines. This effect was matched by a decrease in DNA synthesis, but only moderate induction of apoptosis. Indeed, dasatinib as well as PP2, another SFK inhibitor, strongly induced autophagy that likely prevented apoptosis. However, inhibition of this autophagic response induced robust apoptosis and sensitised lung cancer cells to dasatinib *in vitro* and *in vivo*. Our results provide an explanation for why dasatinib failed in NSCLC clinical trials. Furthermore, our data suggest that combining SFK inhibitors with autophagy inhibitors could provide a novel therapeutic approach in this disease.

## INTRODUCTION

Lung cancer is the principal cancer killer worldwide. Non-small cell lung carcinomas (NSCLC) represent the majority of cases, with adenocarcinomas and squamous cell carcinomas being the two main subtypes. NSCLC is typically resistant to chemotherapy and early surgical resection is the only curative approach. However, most patients present with advanced disease and do not qualify for this procedure, leading to poor 5-year survival (≤10%). Hence, new therapies are urgently needed to improve this prognosis. Targeted therapies, together with adequate patients’ stratification, have become more popular and successful [1] as the dependency of some tumour subtypes on particular molecular events enables the design of selective compounds combining clinical efficacy with better toxicity profiles. These include molecules targeting cell membrane receptors and intracellular signalling molecules [2]. We and others have shown FGFR inhibitors to be promising tools in the treatment of lung cancer [3, 4]. Indeed, squamous cell carcinoma cells commonly show FGFR amplification, making them particularly sensitive to these compounds [4]. However, the applicability of these findings to lung adenocarcinoma was limited [4]. Conversely, EGFR-targeted therapies show promise in a subset of adenocarcinomas with EGFR activating mutations [5]. Unfortunately, patients frequently develop resistance to these compounds and the improvement in overall patient survival is small [6, 7].

The SRC family kinases (SFKs) include nine non-receptor tyrosine kinases: SRC, YES, FYN, FGR, LYN, HCK, LCK, BLK and YRK (in chicken only). Some are ubiquitous (SRC, FYN and YES), while other exhibit more restricted expression patterns [8]. They associate with diverse receptors, such as tyrosine kinase receptors [9], G-protein coupled, steroid and integrin receptors [8] to mediate cell growth, survival, adhesion, migration and angiogenesis [8]. Consequently, these proteins are perceived as prime targets for cancer therapy [10]. Indeed, SFKs are over-expressed/hyper-activated in various malignancies and this correlates with disease progression and poor patient prognosis [10]. The expression and activity of SRC are also found increased in NSCLC [11, 12] with 60–80% of adenocarcinomas and bronchio-alveolar cancers and 50% of squamous cell carcinomas overexpressing this kinase. However, the expression pattern of other SFKs in lung cancer is currently unknown.

Numerous SFK inhibitors have successfully entered clinical trials. One of the most promising compounds, BMS-354825 (aka. Dasatinib) is an ATP-competitive tyrosine kinase inhibitor (TKI) targeting all SFKs [13]. It is successfully used in the treatment of patients with chronic myelogenous leukemia (CML) and Philadelphia chromosome-positive acute lymphoblastic leukemia (Ph+ ALL) [14]. Although, multiple pre-clinical studies indicate that it may be beneficial in the treatment of other cancers including NSCLC, clinical trials have failed to demonstrate benefit in patients [15]. The reasons for this are likely to include inappropriate patient selection and a poor understanding of the biological response to SFK inhibitors [15].

Here, we find that SFKs are overexpressed and hyperactivated in all NSCLC cell lines tested and treatment with SFK TKIs prevents their growth *in vitro*. In particular, downregulation of LYN and/or FYN, that we find overexpressed in primary NSCLC tumours as compared to normal lung, impairs the growth of NSCLC cells. This is clinically relevant as FYN and LYN levels inversely correlate with patient survival with increased LYN being a significant adverse factor in both uni- and multivariate analysis. We show that the effects of dasatinib in NSCLC cells are principally mediated through cell cycle inhibition rather than induction of cell death. Indeed, this TKI induces autophagy in treated cells, preventing the onset of apoptosis. Crucially, inhibition of autophagy sensitises NSCLC cells to dasatinib by promoting apoptosis. In particular, the combination of dasatinib with C1A, a novel orally-available autophagy inhibitor, potentiates tumour regression in our mouse xenograft model. Therefore, this drug combination may represent a novel therapeutic strategy for the treatment of NSCLC patients.

## RESULTS

### SFKs are overexpressed or hyperactivated in lung cancer cells lines

**Figure 1:**
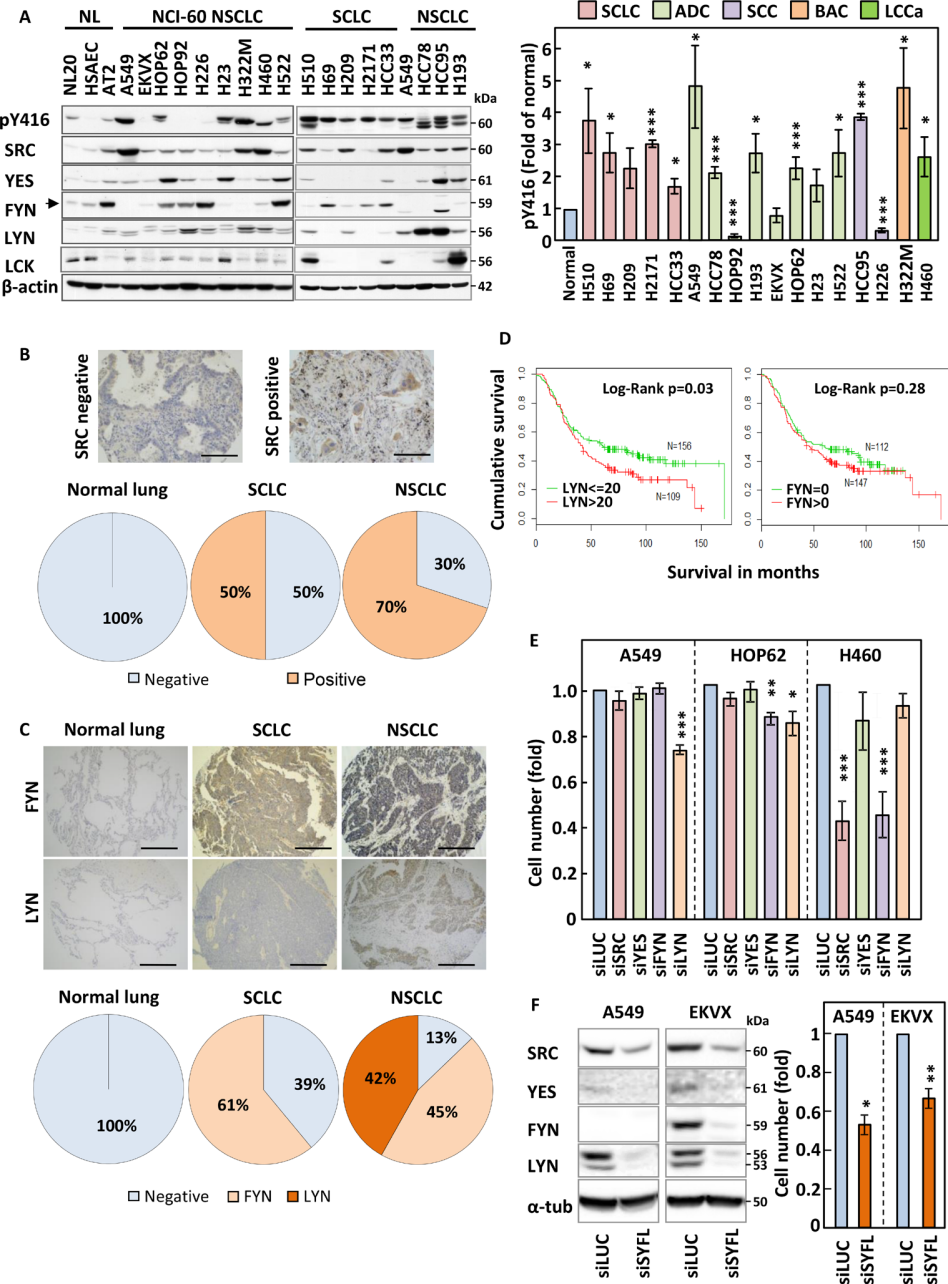
SRC family members are overexpressed in lung cancer (**A**) Expression and tyrosine phosphorylation (Y416) of SRC family kinases (SFKs) in small-cell (SCLC) and non-small cell lung cancer (NSCLC) cell lines as compared to normal lung epithelial cells. Cell protein extracts were analysed by SDS-PAGE/Western blotting for the indicated proteins. Detection of β-actin served as loading control. NL; normal lung immortalised cell lines, NCI-60; cell lines part of the National Cancer Institute panel. (A-right panel) The phosphorylation of SFKs at Y416 was quantified by optical densitometry, normalised to actin levels and represented as fold of levels found in AT2 cells. The data represent the average of three independent experiments ± SEM. (**B** and **C**) Lung cancer and normal lung specimen (*N* = 469 and 61, respectively) were stained either for SRC (B) or for FYN and LYN (C). Representative staining results are pictured in the upper panel and quantification of the proportion of stained specimen is shown in the lower panels. (**D**) Kaplan-Meier survival curve for patients with LYN≤20 and those with LYN>20 (left) and patients with FYN = 0 and those FYN >0 (right). *N* = 284. (**E**) A549, HOP62 and H460 were transfected with pools of 4 siRNA sequences targeting the expression of SRC, YES, FYN or LYN and the effects of this on cell growth was monitored by crystal violet staining. Silencing of Luciferase (siLUC) was used as a negative control. (**F**) A549 and EKVX cells were transfected with siRNA pools targeting SRC, YES, FYN and LYN together (siSYFL). Left panel; cell lysates were analysed by SDS-PAGE/Western blotting for the indicated proteins. Right panel; cell growth was monitored by crystal violet staining. (E and F) Results shown are averagre of three independent experiements performed in quadruplicate ± SEM. Statistical analysis: (A-right panel) Student's *t*-test with Welch correction, (E and F) ANOVA (^*^p < 0.05, ^**^p < 0.01, ^***^p < 0.005).

We first wanted to investigate whether SFKs were overexpressed or hyperactivated in lung cancer cell lines as compared to normal lung epithelial cells. The expression of SFKs or their activation determined by the phosphorylation of a conserved tyrosine residue; Y416 were analysed by SDS-PAGE/Western blotting in 12 non-small cell lung cancer (NSCLC), five small cell lung cancer (SCLC) and three human immortalised normal lung epithelial cell lines of different histological origin: bronchus epithelial cells (NL20), human smooth airways epithelial cells (HSAEC) and type-II pneumocytes (AT2). This revealed that SFKs were hyperphosphorylated in most lung cancer cell lines as compared to their normal counterparts (Figure 1A). Also, one or more SFKs were commonly overexpressed in lung cancer samples as compared to normal lung epithelial cells although this alone could not always account for the increased Y416 phosphorylation. Indeed, while the increase in SFK Y416 phosphorylation in HCC95 may be explained by the total increase in expression of SFKs in this cell line, upregulation of SFKs alone cannot explain the increased Y416 phosphorylation detected for other cell lines such as H69, H322M or A549 (Figure 1A and Supplementary Figure 1A). In particular, SRC was overexpressed in most lung cancer samples in addition to either LYN or FYN. Some cell lines also overexpressed LCK, a SRC family member normally exclusively expressed in the haematopoietic lineage, or YES. The specificity of all antibodies used was confirmed using lysates from cells silenced or not for each individual SFK (data not shown). Taken together, our data suggest that SFKs are both hyperactivated and/or overexpressed in lung cancer cell lines.

### SRC, FYN and LYN are overexpressed in lung cancer patient samples as compared to normal lung tissue

To assess the clinical relevance of our *in vitro* findings, we used tissue microarrays (TMAs) containing 469 lung cancer and 61 normal lung patient samples. These were analysed for expression of FYN and LYN and the specificity of the signal detected by our antibodies confirmed using paraffin embedded cell pellets silenced or not for the corresponding SFK isoform. As an internal control for this study, we additionally stained these samples for SRC, as this SFK was previously shown to be overexpressed in NSCLC [11, 12]. Figure 1B–1C show that while SRC, LYN and FYN were undetectable in normal lung samples, SRC was over-expressed in 50% and 70%, FYN in >61% and 45% and LYN in none and 42% of SCLC and NSCLC samples, respectively. Hence, our *in vitro* expression data for SFKs are mostly representative of the clinical setting and suggest that expression of SFKs may participate to lung cancer progression.

### The expression of LYN correlates with decreased patients overall survival

We next determined whether over-expression of SFKs might impact on prognosis. As SRC expression in lung cancer has previously been examined [16] we focused on the expression of FYN and LYN using two different tissue micro arrays comprising 146 (TMA1) and 138 (TMA2) surgically resected NSCLC cases. Each microarray had a maximum of either 3 or 4 tissue cores per case. FYN and LYN staining was assessed using a 0–300 immunohistochemistry (IHC) scoring system as previously described [17]. The mean IHC score for each patient was used to study the association with survival. Supplementary Table 1 shows the demographic and clinical characteristics of the two TMAs. For simplicity and to increase the power of subsequent analyses we grouped the patients tumours into stage I vs stage II-IV, grades 1/2 vs 3/4 and combined the data from both TMA sets [17]. A restricted cubic spline analysis revealed that an IHC score of 20 provided the optimal cut-off where the hazard starts to increase. In contrast, a linear increase was observed for FYN and no such cut-off point could be demonstrated. Therefore, we use 0 as a cut-off to dichotomise FYN into negative and positive staining (Supplementary Figure 2). Univariate analysis revealed that increasing stage and tumour grade were as expected significantly associated with poor survival (Table 1). In addition, both LYN and FYN staining associated with poor prognosis but only LYN was statistically significant (Table 1 and Figure 1D). After adjusting for age and clinical covariates, the association with LYN became borderline significant (*p* = 0.07, HR (95%CI) = 1.36 (0.98–1.90)) (Table 1).

**Table 1:** Hazard Raito for LYN and FYN in cox survival analysis

	Univariate analysis		Multivariate analysis	
	HR (95%CI)	*P* value	HR (95%CI)	*P* value
**Age (per 1 yr increase)**	0.98 (0.97–1.00)	0.03	0.99 (0.97–1.01)	0.21
**Stage**				
Stage I (*n* = 165)	1		1	
Stage II–IV (*n* = 114)	2.29 (1.69–3.10)	<0.001	2.20 (1.61–3.01)	<0.001
Missing (*n* = 5)	2.25 (0.82–6.14)	0.12	2.98 (1.03–8.65)	0.04
**Grade**				
Grade 1/2 (*n* = 122)	1		1	
Grade 3/4 (*n* = 138)	1.43 (1.05–1.95)	0.02	1.42 (1.01–1.99)	0.05
Missing (*n* = 24)	1.11 (0.63–1.94)	0.72	1.00 (0.55–1.81)	1.00
**Cancer type**				
ADC (*n* = 148)	1		1	
SCC (*n* = 93)	0.87 (0.62–1.20)	0.39	0.83 (0.59–1.16)	0.27
LCC (*n* = 31)	1.03 (0.63–1.67)	0.91	0.77 (0.45–1.31)	0.33
Other (*n* = 12)	0.78 (0.36–1.69)	0.53	0.97 (0.42–2.22)	0.94
**LYN**				
≤20 (*n* = 156)	1		1	
>20 (*n* = 109)	1.39 (1.03–1.88)	0.03	1.36 (0.98–1.90)	0.07
Missing (*n* = 19)	1.11 (0.60–2.04)	0.74	1.09 (0.47–2.50)	0.84
**FYN**				
0 (*n* = 112)	1		1	
>0 (*n* = 147)	1.21 (0.88–1.65)	0.24	1.07 (0.75–1.51)	0.72
Missing (*n* = 25)	1.11 (0.64–1.94)	0.71	1.05 (0.48–2.28)	0.91

### Silencing of LYN, FYN and/or SRC leads to reduced growth of NSCLC cells

Our *in vitro* and immunohistochemistry expression data prompted us to investigate the impact of downregulating SRC, LYN, FYN and YES on the growth of NSCLC cell lines. We selected 3 separate NSCLC cell lines based on their differing pattern of SFK expression (A549, HOP62 and H460 cells) and used siRNAs to target individual SFKs (Supplementary Figure 3A). The three cell lines were differentially sensitive to the silencing of individual SFKs with downregulation of LYN decreasing cell numbers in A549 and HOP62 alone, while FYN silencing inhibited the growth of HOP62 and H460 but not A549 (Figure 1E), consistent with the lack of expression of this isoform in A549 cells (Figure 1A). SRC silencing only hindered the growth of H460 despite being equally expressed in A549 cells. As FYN silencing in H460 led to a decrease in cell number similar to that obtained in response to SRC siRNAs, we tested the effect of combining SRC and FYN silencing in this cell line. Although silencing both SFKs simultaneously led to a further decrease in cell number, this difference was not statistically significant as compared to single SRC knockdown (Supplementary Figure 3B). In addition, the extension of this siRNA study to our complete NSCLC cell panel revealed that some cell lines (eg EKVX and H522) did not show any growth inhibition in response to the downregulation of single SRC family members (Supplementary Figure 3C). This suggests that expression levels for individual SFKs alone cannot predict the sensitivity of NSCLC cells to their targeting. Also, the growth inhibition obtained with the silencing of individual SFK isoforms was poor in A549 and HOP62 cells, implying that the simultaneous targeting of multiple SFKs may be required to achieve an appreciable biological effect. Hence, siRNAs targeting SRC, LYN, FYN and YES were combined, leading to a substantial cell growth inhibition far exceeding that achieved by silencing individual SFK isoforms (Figure 1F). Hence, the simultaneous targeting of multiple SFKs may efficiently prevent the growth of lung cancer cells.

### The pan-SRC kinase inhibitor, dasatinib, inhibits the growth of NSCLC but not SCLC cells

**Figure 2:**
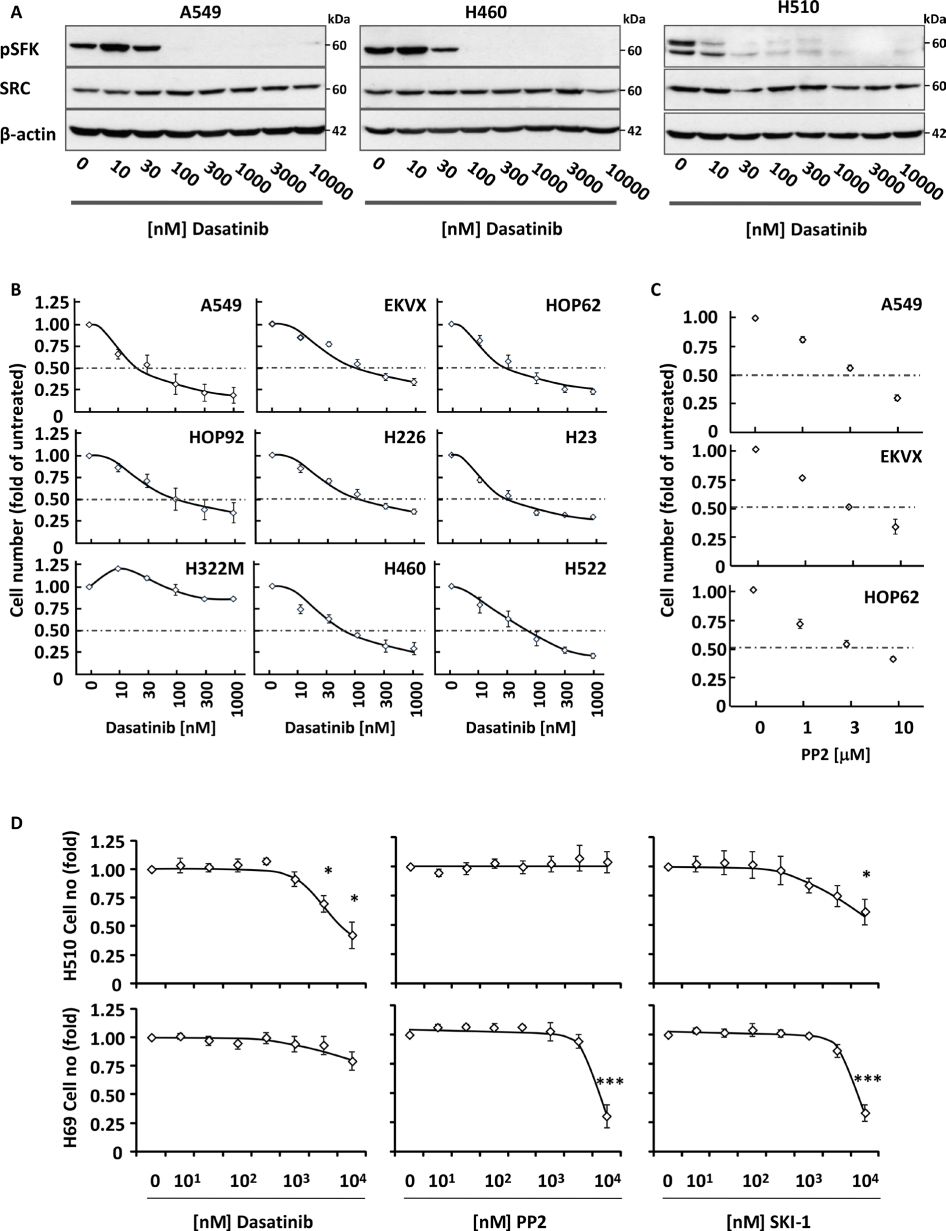
Dasatinib inhibits the growth of NSCLC cells (**A**) Increasing concentrations of dasatinib inhibit autophosphorylation of SRC on Y416 in NSCLC (A549 and H460) and SCLC (H510) cells. Cell lysates were analysed by SDS-PAGE/Western blotting for the indicated proteins. Detection of β-actin served as loading control. (B-D) NSCLC (**B** and **C**) or SCLC (**D**) cells were treated with increasing concentrations of dasatinib (B and D), PP2 (C and D) or SKI-1 (D) and cell growth was monitored by crystal violet staining (B and C) or WST1 assay (D). Graphs represent means ± SEM from three independent experiments performed in triplicate and normalised to control. (D) Statistical analysis was performed using ANOVA (^*^p < 0.05, ^**^p < 0.01, ^***^p < 0.005).

While we demonstrated that siRNA-mediated silencing of SFKs impaired NSCLC cell growth, we wished to investigate whether inhibition of their kinase activity yielded the same biological effect. Several pan-SRC small molecule inhibitors exist including dasatinib, PP2 and SKI-1. Exposure of A549, H460 (both NSCLC) and H510 (SCLC) cells to increasing concentrations of these compounds lead to a dose-dependent inhibition of SFK Y416 autophosphorylation (Figure 2A), demonstrating that all three drugs were active in these cell systems. This translated into a dose-dependent growth inhibition of all but one (H322M) NSCLC cell lines tested with an IC_50_ for dasatinib ≤100 nM, a concentration physiologically achieved in patients treated with this drug [18] (Figure 2B). These results were reproduced using PP2, confirming that the observed effects were a consequence of the inhibition of SFK activity (Figure 2C and Supplementary Figure 4). In contrast, SCLC cells were resistant to dasatinib, with growth inhibition only achieved at supra-physiological concentrations that did not correlate with the suppression of SFK activity in these cells (Figure 2A and 2D). This lack of response extended to the other SFK inhibitors, PP2 and SKI-1 (Figure 2D). Taken together, these data demonstrate that the growth of NSCLC, but not SCLC, cells is sensitive to the inhibition of SFK activity.

**Figure 3:**
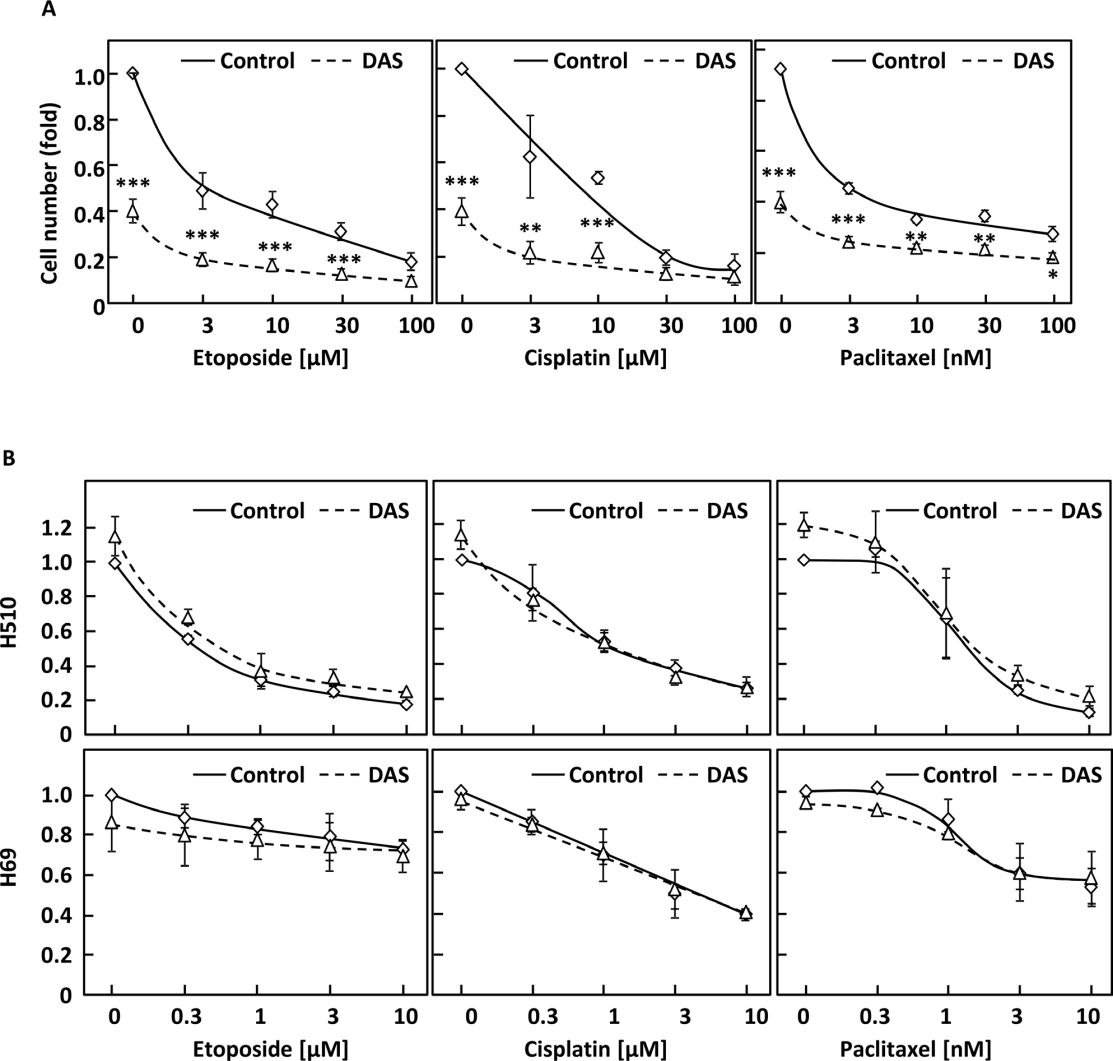
Dasatinib has an additive effect with common therapeutic drugs in NSCLC but not SCLC cells (**A** and **B**) A549 (A) or H510 and H69 cells (B) were treated with or without 30 nM (A) or 1 μM dasatinib (DAS) and increasing concentrations of etoposide, cisplatin or paclitaxel. Cell growth was monitored by crystal violet (A) or WST-1 assay (B) 48 h later. Graphs represent means ± SEM from three independent experiments performed in triplicate and normalised to control. Statistical analysis was performed using ANOVA (^*^p < 0.05, ^**^p < 0.01, ^***^p < 0.005).

We next wished to investigate whether dasatinib sensitised NSCLC cells to chemotherapeutic agents commonly used in lung cancer treatment. A549 cells were treated with a combination of 100 nM dasatinib and increasing concentrations of etoposide, cisplatin and paclitaxel. Figure 3A shows that dasatinib did not synergise with these drugs, but enabled higher total growth inhibition when used in combination with low doses of chemotherapeutic agents. As dasatinib is a well-tolerated drug in the clinic [19], this suggests that this regimen may decrease the overall toxicity of treatment while providing equivalent therapeutic benefit for NSCLC patients. In contrast, no added benefit of this combination over chemotherapeutic drugs used alone was observed in H510 or H69 cells, in agreement with the lack of efficiency of dasatinib in SCLC cell lines (Figure 3B).

### Cell cycle inhibition rather than apoptosis correlates with responsiveness of NSCLC cells to SFKs targeting

**Figure 4:**
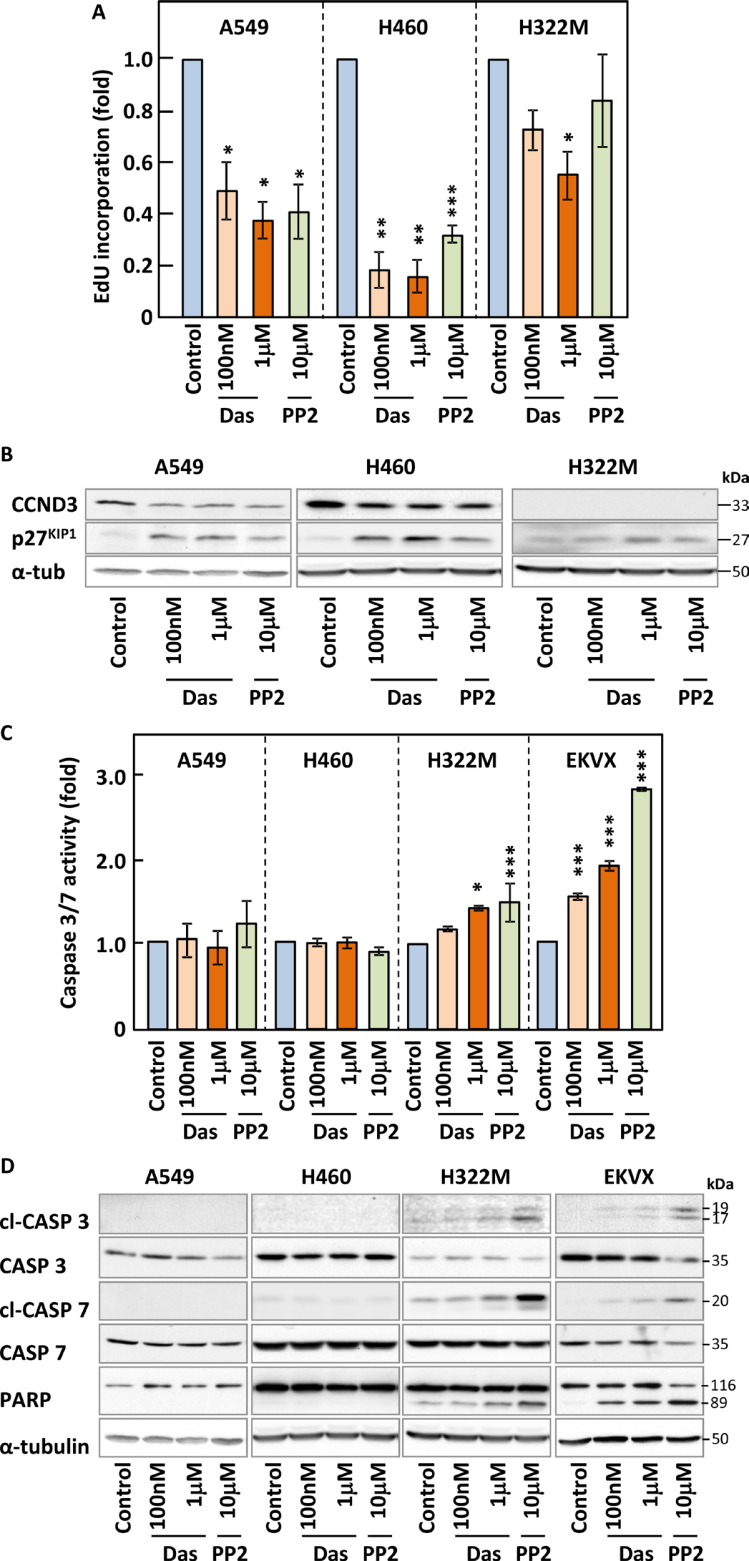
Inhibition SFKs leads to cell cycle arrest but does not trigger efficient apoptosis (**A**–**D**) NSCLC cell lines were treated with the indicated concentrations of dasatinib (Das) or PP2 for 24 h and samples subjected to Edu staining to assess DNA synthesis (A), SDS-PAGE/Western blotting for the indicated cell cycle regulators (B) and apoptotic markers (D) such as cleaved (cl) caspases and caspase 3/7 activity assays (C). Results shown are either representative (B and D) or the average of of a minimum of three independent experiments performed in quadruplicate. Statistical analysis: unpaired two-tailed Student's *t*-test with Welch correction (^*^p < 0.05, ^**^p < 0.01, ^***^p < 0.005).

To highlight the underlying mechanisms responsible for the activity of SFK inhibitors on NSCLC cell growth, we investigated the impact of these compounds on the cell cycle profile and survival of our cell lines. We used EdU incorporation to quantify DNA synthesis by flow cytometry in our panel of NSCLC cells in the presence or absence of dasatinib or PP2. Figure 4A and Supplementary Figure 5 show that these inhibitors decreased DNA synthesis in all cell lines where they induced growth inhibition. This correlated with increased expression of the cell cycle inhibitor p27^KIP1^ and a corresponding decrease in CCND3, a protein involved in G1-S transition, in A549 and H460 cells (Figure 4B and Supplementary Figure 6A). In contrast, dasatinib or PP2 did not modulate DNA synthesis or the expression of cell cycle regulators in H522M cells at physiologically meaningful concentrations (Figure 4A–4B) consistent with the corresponding lack of growth inhibition obtained in this cell line.

We also investigated whether these inhibitors induced apoptosis by monitoring caspases 3 and 7 activation using activity-based assays (Figure 4C and Supplementary Figure 6B) and Western blotting-based detection of their cleavage as well as that of one of their substrates, PARP (Figure 4D). Very low (≤1.5 fold) or no induction of caspase activity was obtained in all NSCLC cell lines treated with either compounds and this resulted in the poor cleavage of PARP, except in EKVX cells. Moreover, differences in apoptosis levels among our cell lines did not correlate with their responsiveness to the inhibitors. While induction of apoptosis was not a major response to these drugs, other cell death pathways might alternatively be involved. Hence, we monitored changes to the membrane permeability of all our cell lines to propidium iodide in response to dasatinib and PP2 as a marker of necrosis (Supplementary Figure 6C and data not shown). Once again, little cell death was detected as a result of drug treatment. Taken together, our data suggested that a decrease in DNA synthesis was mostly responsible for the growth inhibitory effects of SFK inhibitors in NSCLC cells. However, an additional pathway previously involved in cell death, autophagy, may still be involved in the inhibitory effects of these compounds on our cell lines.

### Inhibition of SFKs induces autophagy

**Figure 5:**
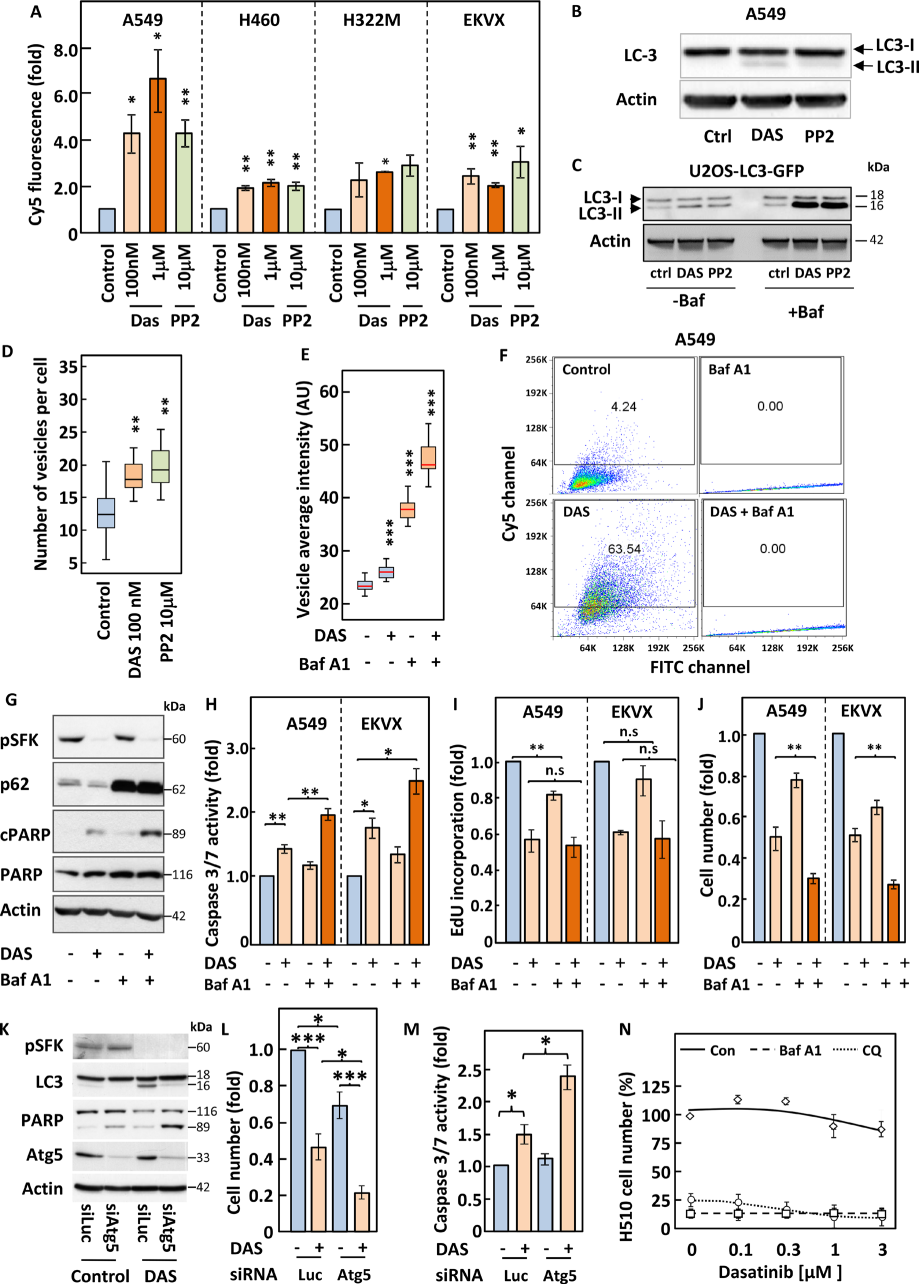
Inhibition of autophagy induced by SFKs targeting sensitise cells to dasatinib (**A**–**N**) The indicated cell lines were pre-treated with or without 3 nM bafilomycin A1 (Baf A1) for 2 h (D–J and N) and incubated with the indicated concentrations of dasatinib (Das) or PP2 (A, C and N) or 100 nM dasatinib (B–M) for 16 h. (A and F) Cells were incubated with 1 mg/ml acridine orange for 15 min prior to analysis by flow cytometry for red and green fluorescence, corresponding to lysosomal acidicity and DNA content, respectively. (B, C, G and K) Lysates from A549 cells (B, G and K) or U2OS cells stably expressing the LC3-GFP fusion protein (U2OS-LC3-GFP) (C) were analysed by SDS-PAGE/Western blotting for the indicated proteins. Detection of Actin served as a loading control. (D and E) U2OS-LC3-GFP cells were fixed, stained with DAPI and images were acquired using an ImageXpressMicro high-content microscope. (D) Representative image acquired at x20. Blue; nuclei, green; autopagosomes. (E) 30 images per condition, each with an average of 100 cells, were analysed using Metamorph and SPSS. A distribution of vesicles average intensity is represented as box and whisker plots. Box represents 75% and side bar 12.5% of the cell population. Line indicates the median. (H–J) The indicated cell lines were subjected to a luminometric caspase3/7 activity assay (H), (I) an EdU-based DNA synthesis (I) or a crystal violet-based cell growth assay (J). (L–M) A549 cells transfected with or without Atg5-targeting siRNA pools were subjected to a crystal violet-based cell growth assay (L) or a luminometric caspase3/7 activity assay (M). A luciferase (Luc)-targeting siRNA pool was used as control. (N) H510 cells were subjected to a WST1 growth assay. Statistical analysis (A) ANOVA, (E) Kruskal Wallis test and (H–J and L–M) Student's *t*-test. ^*^p < 0.05, ^**^p < 0.01, ^***^p < 0.005.

Autophagy has been suggested to mediate either cell death or cell survival depending on the cell system considered [20]. We therefore investigated whether inhibition of SFKs modulated autophagy in our NSCLC cell lines. To this end, we used three distinct complementary approaches. First, we assessed the formation of autophagolysosomes by monitoring changes in the fluorescence emission of acridine orange that occur following acidification of autophagosomes through fusion with lysosomes. Second, we assessed the post-translational modification of the protein LC3 (LC3-I to LC3-II) that accompany its inclusion into the membrane of autophagosomes. Third, we used the well-documented U2OS-based cell line expressing a fluorescently-tagged version of LC3 (U2OS-LC3-GFP) [21] in which changes in autophagy can be monitored by quantifying the appearance of fluorescent cytoplasmic vacuoles. These three methods revealed that inhibition of SFKs with either dasatinib or PP2 induced autophagy in both NSCLC and U2OS cells (Figure 5A–5D and Supplementary Figure 7). Indeed, flow cytometry analysis of acridine orange-stained NSCLC cells showed a shift in their emitted fluorescence from green (FITC) to red (Cy5) in response to treatment with either compound (Figure 5A and Supplementary Figure 7). This correlated with post-translational modification of LC3 in A549 and U2OS cells as shown by the appearance of a lower molecular weight band for this protein by Western blotting (Figure 5B–5C) and the appearance of cytoplasmic LC3-GFP containing vacuoles in U2OS cells (Figure 5D–5E and Supplementary Figure 8A). In light of the controversy on the role of autophagy in cell survival, we proceeded to inhibit this pathway to assess its impact on the response to dasatinib. For this purpose, we used two compounds that target different stages of the autophagic process; chloroquine and Bafilomycin A1. Bafilomycin A1 prevents maturation of autophagosomes, leading to their cytoplasmic accumulation. This was readily detected in U2OS-LC3-GFP cells, where treatment with this compound increased the intensity, number and size of autophagosomes and the processing of LC3 (Figure 5C–5E and Supplementary Figure 8B). This drug also prevented the shift in the fluorescence emission of acridine orange in A549 cells as it impairs the fusion of autophagosomes with lysosomes (Figure 5F). The inhibition of autophagy by Bafilomycin A1 potentiated the induction of apoptosis in response to dasatinib as demonstrated by increased caspases activation of 3 and in A549 and EKVX cells and the cleavage of PARP in A549 cells (Figure 5G–5H). In contrast, Bafilomycin A1 had no effect on the dasatinib-induced inhibition of DNA synthesis in A549 or EKVX cells (Figure 5I). The increase in apoptosis exacerbated the growth inhibitory effects of dasatinib in both cell lines (Figure 5J). A comparable enhancement of apoptosis and growth inhibition were obtained when chloroquine was used in combination with dasatinib, supporting the idea that the inhibition of autophagy is underlying this improved response (Data not shown). This was further confirmed in A549 cells where siRNA-mediated silencing of ATG5, a protein essential to the autophagic process [22], reproduced the effects of bafilomycin A1 and chloroquine (Figure 5K–5M). We have shown that SCLC cells are resistant to dasatinib and wondered whether an increase in baseline levels of autophagy in these cells may account for this. Indeed, Western blot analysis of H510 and H69 cell lysates for LC3 post-translational modification revealed that these two cell lines show far higher levels of background autophagy than A549 or U2OS cells (Supplementary Figure 9A). Hence we hypothesised that treatment of these SCLC cell lines with bafilomycin A1 or chloroquine may sensitise them to dasatinib. Figure 5N shows that treatment with either autophagy inhibitor led to massive growth inhibition in H510 cells above which additional effects of dasatinib could not be assessed. Similar results were obtained for H69 cells (Supplementary Figure 9B). Taken together, our data suggest that autophagy is a pro-survival mechanism in SFK TKI-treated NSCLC cells that prevents the induction of apoptosis by these compounds.

### Inhibition of autophagy enables dasatinib to target tumour growth *in vivo*

**Figure 6:**
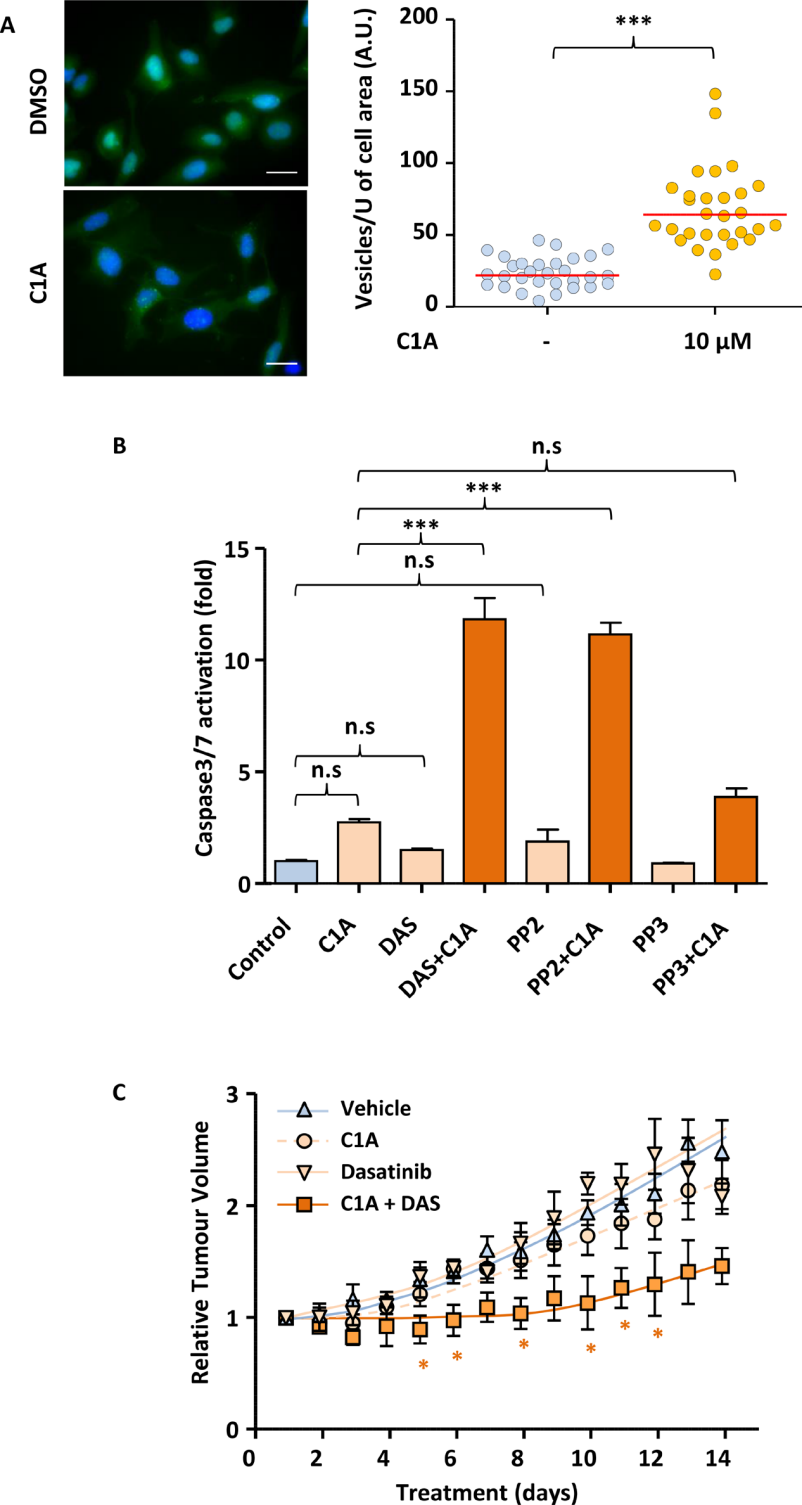
Autophagy inhibition enables dasatinib to reduce tumour volume in A549 mice xenografts (**A**) C1A inhibits autophagy *in vitro*. U2OS-LC3-GFP cells were treated or not with 10 μM C1A for 24 h and the accumulation of autophagocytic vesicles imaged by fluorescent microscopy and quantified in Image J. Images shown are representative of *n* = 30/condition. (**B**) C1A promotes the induction of apoptosis by dasatinib *in vitro*. A549 cells treated with or without C1A (10 μM), dasatinib (1 μM), PP2 (10 μM) or PP3 (10 μM) for 24 h and subjected to a luminometric caspase3/7 activity assay. (**C**) Co-administration of C1A and dasatinib prevents A549 xenografts tumour growth in nude mice. A549 cells were injected subcutaneously in nude mice and treatment initiated when tumours reached 50-100 mm^3^ with or without 20 mg/kg C1A and 75 mg/kg dasatinib by daily oral gavage for 2 weeks. Tumour volumes were determined by caliper measurement. *N* = 5 per condition. (A–C) Results are representative of experiments performed in triplicate. Statistical analysis by ANOVA with ^*^p < 0.05, ^**^p < 0.01, ^***^p < 0.005.

Our *in vitro* results suggest that inhibition of autophagy renders NSCLC cells sensitive to SRC inhibition. We next wished to assess if this could be translated to the *in vivo* setting. While Bafilomycin A is a very efficient autophagy-blocking agent *in vitro*, it is not deliverable orally. Also, while chloroquine and its derivatives are being used as an autophagy inhibitor in cancer clinical trial, it has significant toxicity [23]. We recently identified a novel autophagy inhibitor, C1A that can safely be administered orally in animals and may show clinical potential. Indeed, daily oral administration of 20 mg/kg C1A to nude mice did not cause any weight loss as compared to vehicle only (Supplementary Figure 10A). Figure 6A shows that just as for Bafilimycin A, treatment of U2OS-LC3-GFP cells with C1A led to the accumulation of autophagosomes due to the failure of their fusion with lysosomes. As a consequence of this, co-administration of C1A enabled efficient caspase activation in A549 treated with dasatinib or PP2 (Figure 6B). This was linked to the ability of these compounds to inhibit SRC proteins, as no potentiation of caspase activation was achieved following treatment of A549 cells with a combination of C1A and the non-SRC targeting PP2 analogue, PP3 (Figure 6B). We therefore tested whether the combination of C1A and dasatinib was effective in an A549 xenograft mouse model. Nude mice were injected with A549 cells subcutaneously and the animals randomised once tumours reached 50–100 mm^3^ in any dimension. Animals were then treated daily with or without 75mg/kg dasatinib and 20 mg/kg C1A by oral gavage. Figure 6C shows that while dasatinib or C1A had no effect when administered alone, combination of the two compounds led to a profound inhibition of tumour growth. This was associated with the efficient onset of intratumoural apoptosis in animals treated with this combination, as suggested by the increased cleavage of Lamin B, a downstream caspase substrate (Supplementary Figure 10B). Taken together, our results demonstrate that autophagy inhibition may sensitise NSCLC to SRC inhibition in the clinic.

## DISCUSSION

Targeted therapeutic agents are relatively novel tools in the fight against cancer. Their efficacy often depends on the overexpression/hyperactivation of the intended protein target and the addiction of the cancer cells on associated downstream signalling events. While inhibition of cell proliferation may help control disease progression, induction of cell death is the preferred outcome of anti-cancer therapies.

The expression or kinase activity of SRC were previously shown increased in NSCLC [11, 12], leading to multiple clinical trials being initiated in this disease using SRC TKIs, including dasatinib [24]. However, these invariably showed poor results [15] for reasons that remained unclear. Hence, we decided to investigate both the validity of this therapeutic approach in NSCLC as well as possible reasons for the observed therapeutic failure. We found that lung cancer cells generally overexpressed one or more SFK as compared to their normal counterpart both *in vitro* and *in vivo* (Figure 1A–1C). This was associated with increased SFK activity in lung cancer cells (Figure 1A). In particular, SRC together with YES, FYN and LYN were often found co-overexpressed in our cancer cell lines and tissue sections (Figure 1A–1C).

While the overexpression/hyperactivation of SRC is associated with poor prognosis in patients suffering from various cancers [10], similar studies for LYN and FYN led to varied conclusions. For example, FYN and LYN were found overexpressed in renal carcinoma but no link between these isoforms and patient survival was established [25]. Similarly, while LYN was involved in the tumourigenesis of breast cancer [26], its expression was not linked to changes in patients survival [27]. In contrast, in colorectal cancer, LYN overexpression was positively correlated with tumour grade, stage, lymph node involvement/distant metastasis and lower patient survival. However, multivariate analysis revealed that this SFK was not an independent prognostic factor in this disease [28]. Conversely, high LYN expression was associated with good prognosis in ovarian cancer [29]. As for FYN, its increased expression correlated with shorter failure-free survival in Hodgkin lymphoma [30], but downregulation of its activity correlated with disease progression in neuroblastoma [31]. FYN was also found overexpressed in NSCLC tumours as compared to normal tissue [32], but the prognostic impact of this was not investigated. Hence, we decided to assess the impact of LYN and FYN overexpression on NSCLC patients’ survival.

The observation that LYN and FYN are widely overexpressed NSCLC tumours while being undetectable in normal lung tissue is consistent with the previously suspected involvement of these SFKs in transformation and tumourigenesis [33–35]. Also, the impact of LYN and FYN on NSCLC patients’ survival may be linked to their ability to regulate biological process associated with cancer progression, such as resistance to apoptosis and metastasis. Indeed, LYN activity associates with resistance to drug-induced apoptosis [36, 37]. Moreover, these SFKs contain an N-terminal region that, when cleaved by caspases, results in their increased activation, re-localisation and pro-survival function [37, 38]. Part of the role of FYN in pro-survival signalling occurs through up-regulation of BCL-XL [39], an anti-apoptotic protein that we implicated in lung cancer drug resistance [40, 41]. In addition to regulating cell death, FYN and LYN participate to cancer cell invasion and metastasis [15, 42].

Our results are consistent with an important role of LYN and FYN in NSCLC biology. Downregulating these kinases *in vitro*, separately or in combination, decreased the growth of NSCLC cells (Figure 1E–1F). This together with the increased SFK activity seen in these cell lines suggested that SRC TKIs may be efficient therapeutic agents against lung cancer. Indeed, we found that small-molecule-based inhibition of SFKs lead to decreased proliferation of NSCLC cells. However, in agreement with data published for other cancer cell types [43, 44], very little apoptosis was observed in response these compounds. This translated into the inability of dasatinib monotherapy to prevent tumour growth in a NSCLC mouse xenograft model (Figure 6C), reminiscent of results obtained in the clinic [15]. We demonstrate that the lack of apoptosis onset by dasatinib is linked to the induction of autophagy by this compound. Autophagy has previously been involved in resistance of various cancers to therapy [45]. Similarly, we find that inhibition of autophagy, either through downregulation of autophagic core machinery components or using small-molecule inhibitors, sensitises NSCLC cells to dasatinib. This occurs through promotion of dasatinib-induced apoptosis and enables dasatinib to prevent tumour growth in our xenograft model. Conversely, SCLC cell lines that have high baseline levels of autophagy were inherently resistant to SRC TKIs despite background SFK hyperactivation.

Taken together, our data suggest that the clinical failure of SRC TKIs in the treatment of NSCLC is due to the induction of intratumoural autophagy by these compounds that prevents apoptosis. The inhibition of autophagy in combination with classical and targeted chemotherapy is currently the subject of numerous clinical trials in lung and other cancers [46]. In particular, hydroxychloroquine has been used in a lung cancer trial where it was co-administered with the EGFR TKI, erlotinib [47]. However, ocular, gastrointestinal and skin toxicity are major side-effects of this compound in the long-term treatment of rheumatoid arthritis and lupus erythematosus [23]. Hence, this compound is not likely to represent a viable long-term solution in the clinic. Similarly, bafilomycin A, while showing low toxicity in animal, is not orally available. We have recently identified a novel HDAC6 targeting agent, C1A [48], which also efficiently inhibits autophagy by preventing the maturation of autophagosomes. This compound can be delivered orally and does not show general toxicity in mice when administered daily at an effective dose of 20 mg/kg [48] (Supplementary Figure 10A). Co-administration of this compound with dasatinib is efficient at preventing the growth of NSCLC xenograft in mice, at least partly through increased intratumoural apoptosis (Supplementary Figure 10B), and this combination may provide clinical benefit to NSCLC patients. While we tried to investigate whether autophagy inhibition may also sensitise SCLC cells to dasatinib, this was prevented by the exquisite sensitivity of SCLC cells to inhibition of the autophagic pathway. Indeed, exposure of H510 or H69 cells to bafilomycin A or chloroquine led to massive cell death beyond which the effects of dasatinib were undetectable. This finding may suggest that SCLC tumours may be far more sensitive than other types of lung cancer to autophagy inhibitors, a possibility worth testing in a disease where novel therapies are urgently needed to improve bleak survival rates.

## MATERIALS AND METHODS

### Materials

See Supplementary Materials and Methods.

### Cell lines and culture

All cell lines (except U2OS- LC3-GFP, AT2 and A549-H2B-GFP) were from the ATCC. P2G (AT2) cells were provided by Prof Terry Tetley (NHLI, Imperial College London). U2OS cells stably over-expressing LC3-GFP were a gift from Dr Sharon Tooze (CRUK-LRI, London). A549 cells stably expressing H2B-GFP were manufactured in our lab. All lung cancer cell lines were grown in RPMI1640, U2OS and U2OS-LC3-GFP in DMEM and AT2 cells in DCCM-1 medium (Invitrogen). Media were supplemented with 10% foetal calf serum (FCS), 2 mM L-glutamine, 50 units/ml penicillin and 50 μg/ml streptomycin and cells grown in 5% (RPMI/DCCM1) or 10% (DMEM) CO2, 37°C. Prior to experiments NSCLC and U2OS cells were grown in starvation medium (RPMI1640/DMEM) containing 0.5% FCS and 2 mM L-glutamine for 6 hours (Crystal Violet and Caspase assays) or over-night and SCLC cells were grown for 3 days in SITA (RPMI-1640, 5 μg/ml insulin, 10 μg/ml transferrin, 30 nM sodium selenite and 0.25% (w/v) bovine serum albumin (BSA)). U2OS-LC3-GFP cells were grown with 0.5 mg/ml (w/v) G418.

### SDS-PAGE/Western blotting

Cells were lysed in RIPA buffer (50 mM Tris-HCl pH 7.4, 150 mM NaCl, 1% Triton X-100, 1% sodium deoxycholate, 0.1% SDS, 1 mM EDTA; protease inhibitors (Roche complete tablets), 5 mM sodium flouride, 1 mM sodium orthovanadate, 50 mM β-glycerophosphate). Samples were kept on ice for 30 min and cleared by centrifugation at 16000 g for 10 min. Lysates were boiled for 5 min in Laemmli buffer (0.5 M Tris, pH 6.8, 4% SDS, 10% glycerol, bromophenol blue), subjected to SDS-PAGE and proteins transferred onto nitrocellulose (iBlot System, Invitrogen) prior to probing with the relevant antibodies.

### Immunohistochemistry

Commercial TMAs LC1006, LC1201, LC10010 containing normal lung tissue, NSCLC and SCLC samples were purchased from Biomax, Inc (Rockville, USA).

TMA1 contained 151 resected NSCLC tumours (ADC, SCC and LCC) each represented by three 1 mm cores. Histopathological data on tumour type, grade, lymph node status and stage together with clinical data including age, performance status, time to relapse and overall survival were recorded. After excluding 5 patients with missing follow-up time and event, 146 patients were included in the analysis.

TMA2 contained 138 specimens of resected NSCLC (ADC and SCC) tumours each represented by four 1mm cores. The dataset contains information about overall survival, age, NSCLC type and tumour grade and stage.

For staining procedure, please see Supplementary Material and Methods.

Receiver operating characteristic (ROC) curve was used to plot the sensitivity against the false positive rate of FYN/LYN in predicting the 2-year and 5-year survival. Those who were censored before 2 years and 5 years of follow-up were excluded from the analyses for 2-year and 5-year, respectively. The areas under the ROC curves are less than 0.6 and thus FYN and LYN is less likely to distinguish between patients who can survive to 2/5 years.

The primary outcome of this study is overall survival (OS), defined as time from surgery to death resulting from any cause. Patients were censored at last date of follow-up if they were alive at the last follow-up. Kaplan-Meier curves were plotted to compare the OS between patients with different ICH scores of LYN/FYN. A multivariate Cox proportional hazards model was used to estimate the independent association between FYN/LYN and OS adjusting for clinical and demographic factors. Hazard ratio and its 95% confidence interval (CI) were reported. A *P* value of less than 0.05 was considered statistical significant. All analyses were performed using R version 3.0.2 and the package “rms” (biostat.mc.vanderbilt.edu/rms).

### siRNA transfection

Cells were transfected with 25 nM siRNA (pool of four independent sequences/target) using DharmaFECT II (Dharmacon) according to the manufacturer's instructions. Cells were then incubated for a further 48 h to allow for target downregulation. Luciferase-targeting siRNAs were used as control.

### WST-1 assay

SCLC cells grown for 3 days in SITA were re-suspended in fresh SITA and plated in 96-well plates at 15000 cells/well and used for experiments 2 h later. Cells were grown in the presence of drugs for either 7 (growth assay) or 4 days (TKI+chemotherapy) and cell viability assessed using the WST-1 assay (Calbiochem) according to the manufacturer's protocol. Absorbance was measured at 450 nm with a reference filter at 620 nm.

### Crystal violet staining

Cells were fixed in 4% paraformaldehyde and stained in 0.02% crystal violet solution for 15 min. Plates were washed in water and air-dried. Crystal violet-precipitates were solubilised in 10% (v/v) acetic acid (30 min, room temperature, gentle shaking) and absorbance at 595 nm measured.

### Caspase 3/7 activity assay

The Caspase-Glo 3/7 Assay (Promega) was performed according to the manufacturers’ instructions. Briefly, 100 μl of Caspase-Glo 3/7 reagent was added per 96-well and luminescence measured after 15 and 60 min of incubation at room temperature (PHERAstar microplate reader).

### EdU incorporation assay

EdU incorporation assay (Click-iT^™^ EdU Kit; Invitrogen) was performed according to the manufacturers’ instructions. Samples were analysed by flow cytometry (BD FACS Canto 1) and data analysed using FlowJo.

### Acridine orange staining

Acridine orange was added to the growth medium to a final concentration of 1 mg/ml for 15 min prior to cell trypsinisation. Cells were washed once in PBS and immediately analysed by flow cytometry (BD FACS Canto-1). Data were analysed using FlowJo.

### Autophagy monitoring using fluorescent microscopy

U2OS LC3-GFP cells were starved in 0.5% FCS/DMEM for 4 h, pre-treated or not with 3 nM bafilomycin A1 for 2 h and treated with 100 nM dasatinib for an additional 16 h. Alternatively, U2OS LC3-GFP cells were transfected with 75 nM of the relevant siRNA using Dharmafect 2 and incubated for 48 h at 37°C, 10% CO_2_. Cells were then fixed in 4% paraformaldehyde/PBS, and stained with DAPI to visualise nuclei. 60–90 fields of view were acquired per condition using the ImageXpress Micro (Molecular Devices) and analysed using Metamorph (Molecular Devices) for numbers and characteristics of the LC3-GFP foci. Box-and-whiskers plots were generated in SPSS. Statistical analysis was performed using the Kruskal–Wallis test.

### Animal experiment

A549 cells (5 × 10^6^) were injected subcutaneously in 100 ml volumes into the flanks of female nu/nu-BALB/c athymic nude mice (Harlan). Tumour measurements were performed daily by caliper and volumes were calculated using the formula (length (mm)) x (width (mm)) x (width (mm)) x π/6. When tumours reached a volume of 50–100 mm^3^, treatment was initiated with or without dasatinib (75 mg/kg) and C1A (20 mg/kg) by daily oral gavage. Throughout the 14-day treatment period, animal weights was determined daily.

## SUPPLEMENTARY MATERIALS FIGURES AND TABLE


