# Supplementary material for: Targeting autophagy sensitises lung cancer cells to Src family kinase inhibitors
Source: Oncotarget. 2018 Jun 8;9(44):27346–62. doi: 10.18632/oncotarget.25213 (PMC6007948; doi:10.18632/oncotarget.25213)
Supplement: Supplementary file 1 [file oncotarget-09-27346-s001.pdf]

Targeting autophagy sensitises lung cancer cells to Src family kinase inhibitors

SUPPLEMENTARY MATERIALS

Materials

Dasatinib was a gift from Bristol-Myers Sqibb. FGF-2, etoposide, cisplatin, rapamycin, bafilomycin A1, acridine orange and WST-1 assay were from Merck Biosciences Ltd (Nottingham, UK). The EdU kit was from Invitrogen (Oregon, USA) and the Caspase 3/7 Glo assay from Promega (Madison, USA). All other reagents were from Sigma-Aldrich (Dorset, UK). Antibodies against Phospho-SFKs (Tyr416) (#2101), YES (#2734), FYN for Western blotting (#4023), LYN for IHC (#2796), BLK (#3262), p27KIP1 (#2552), Cyclin D3 (#2936), Cleaved caspase 3 (#9661), Caspase 3 (#9662), Cleaved caspase 7 (#9491), Caspase 7 (#9492), PARP (#9542), ATG5 (#2630), SQSTM1/p62 (#5114) were from New England Biolabs (Hertfordshire,UK), SRC for IHC (#2109), FYN for IHC (sc-73388), LYN for Western blotting (sc-15), LCK (sc-433), HCK (sc-72) were from Santa Cruz Biotechnology (Heidelberg, Germany), LC3 (0231-100) was from NanoTools (Teningen, Germany), α-tubulin (T5168), β-actin (A5441) from Sigma-Aldrich (Dorset, UK) and SRC for Western blotting Millipore (California, USA).

Immunohistochemistry

For antigen retrieval, sections were rehydrated in graded alcohols, microwaved at 900 W for 20 min in Citrate buffer (pH6) and cooled to room temperature before immunostaining. Endogenous peroxidase activity was suppressed by incubation with 3% H_2O_2 for 5 min. SRC, FYN and LYN antibodies (1:100 dilution) were applied for 1 h at room temperature prior processing with Polymer-HRP Kit with Diaminobenzidine development and Mayer haematoxylin counterstaining. Cytoblocks from a cell line positive for the required SFKs were used as positive controls. Negative controls were obtained by omitting the primary antibody and by staining U2OS cells RNAi-depleted for SRC, FYN or LYN.

Each core was assigned an IHC score, calculated by multiplying the percentage of positive cells (0%–100%) and the intensity of staining (0–3, corresponding to negative, weak, moderate and strong positivity). The range of possible scores was thus 0 to 300.

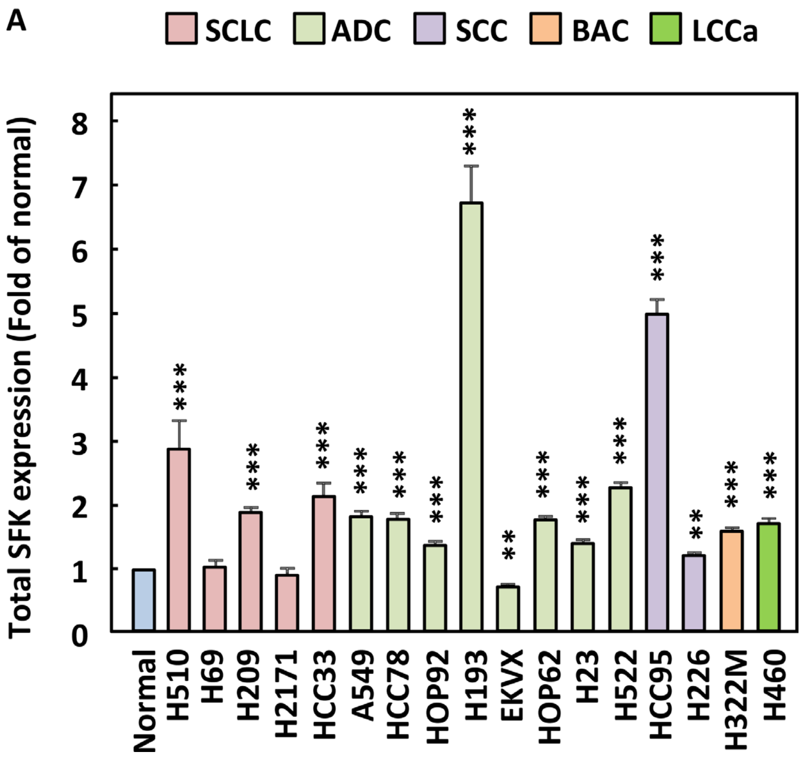

Supplementary Figure 1: SFKs are overexpressed in the majority of lung cancer cell lines as compared to normal lung epithelial cells. Western blotting for SFKs expression in the indicated cell lines, such as those presented in Figure 1A, were quantified by optical densitometry and presented as a bar graph of the mean ± SEM from three idenendent experiments. Values obtained for each SFK was added for individual cell line, the sums corrected by the value obtained for the corresponding β-actin loading control and the corrected sums normalised to the average of the sums obtained for the normal lung epithelial cell lines. Statistical analysis: Student's *t*-test with Welch correction (n.s. $p > 0.05$, $^{**}p < 0.01$, $^{***}p < 0.005$).

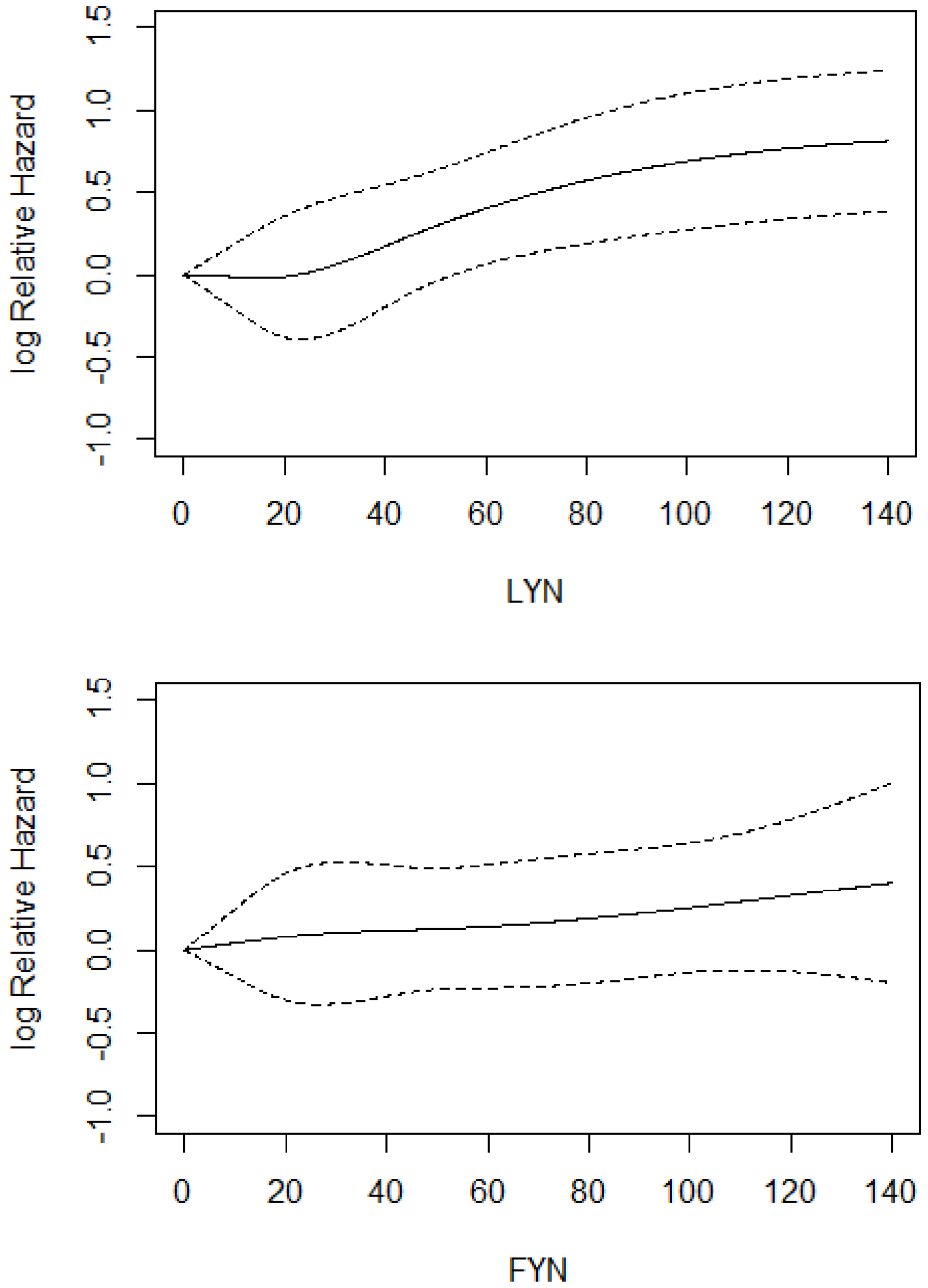

Supplementary Figure 2: Restricted cubic spline for LYN and FYN after combining the two TMA sets. The log relative hazard and its confidence interval were plotted for FYN and LYN as continous scores using restricted cubic spline with IHC score of 0 as reference.

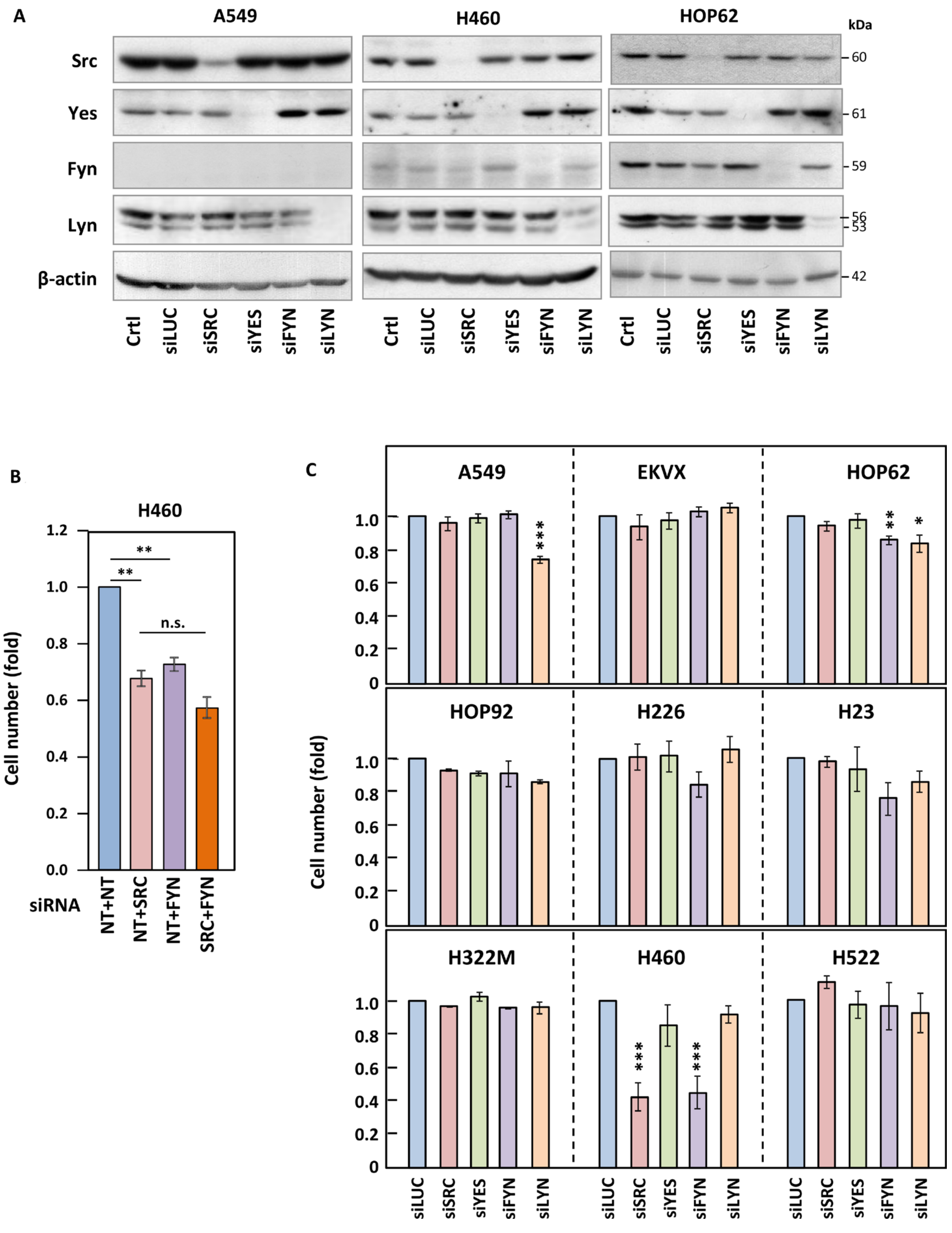

Supplementary Figure 3: siRNA-mediated silencing of single SFK members rarely lead to decreased NSCLC cell growth. The indicated cell lines were treated with (si) or without (Ctrl) siRNAs targeting SRC, YES, FYN, LYN or Luciferase (LUC) used as a negative control. (**A**) Cell lysates were analysed by SDS-PAGE/Western blotting for the indicated proteins. Detection of β-actin was used as a loading control. Blots are representative of three experimental repeats. (**B–C**) Changes in cell growth was assessed 72 h post-transfection using crystal violet staining. Results are means ± SEM from three independent experiments performed in triplicate and normalised to LuciferasesiRNA-treated control cells. Statistical analysis: unpaired, two-tailed Student's *t*-test ($^{*}p < 0.05$, $^{**}p < 0.01$, $^{***}p < 0.005$).

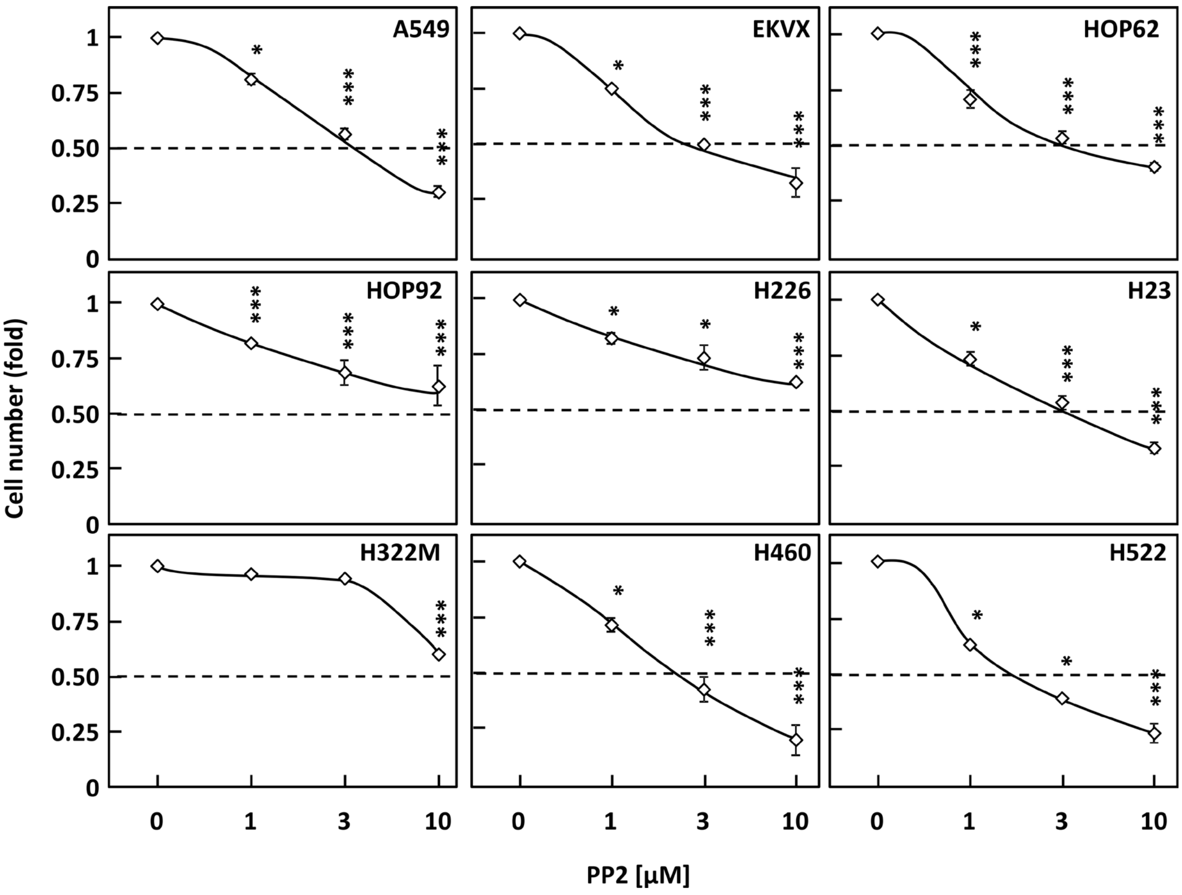

Supplementary Figure 4: Treatment with PP2 inhibits the growth of NSCLC cells. Indicated NSCLC cell lines were treated with or without increasing concentrations of PP2 for 3 days. Cell growth was assessed using crystal violet staining. Results are means ± SEM from at least three independent experiments performed in triplicate. Values are expressed as fold change over untreated (DMSO only) controls. Statistical analysis: Student's *t*-test ($^{*}p < 0.05$, $^{**}p < 0.01$, $^{***}p < 0.005$).

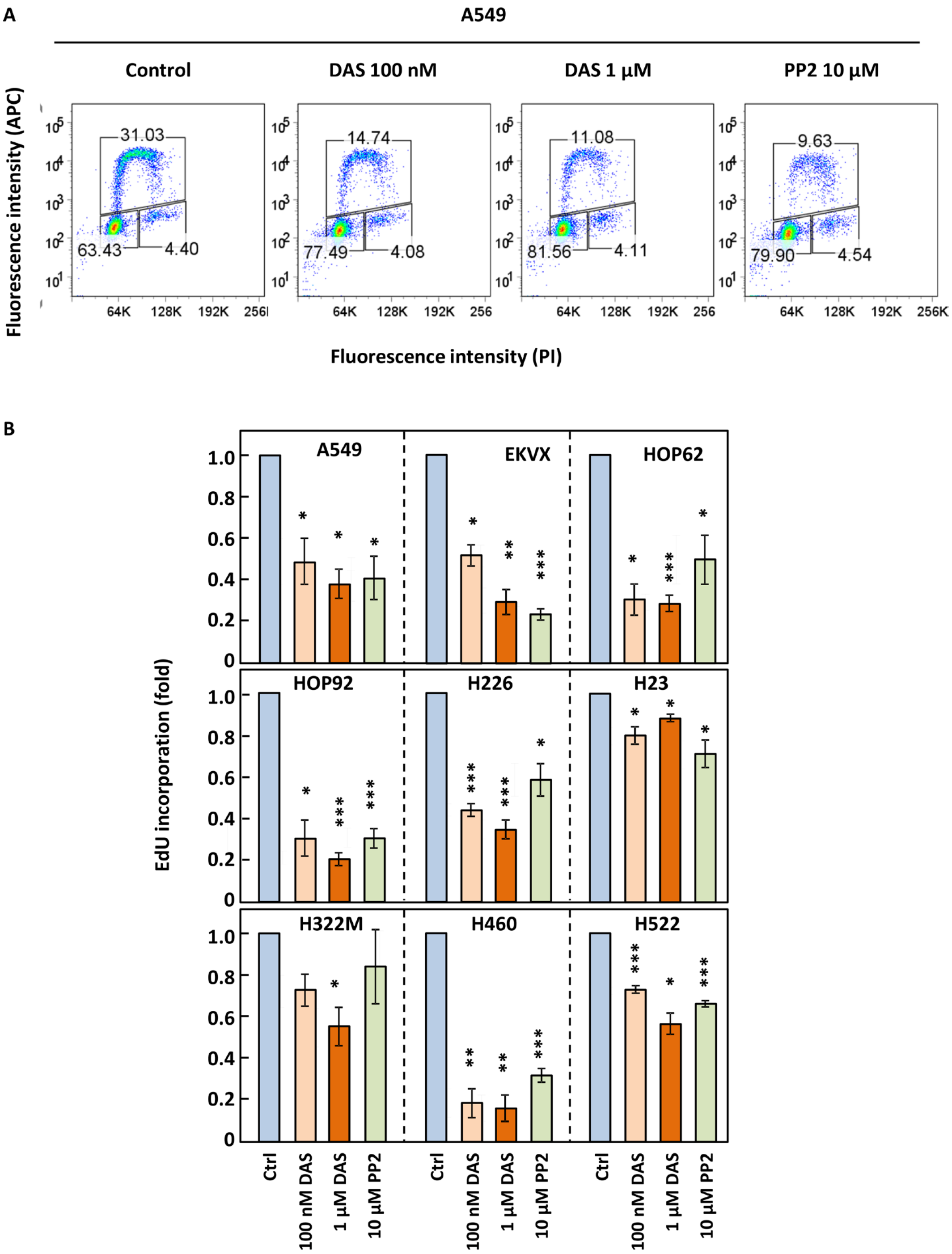

Supplementary Figure 5: SFKs inhibition leads to decrease in DNA synthesis. The indicated cell lines were treated for 24 h with or without dasatinib (DAS) or PP2 at the indicated concentrations. Treatment with DMSO alone was used as control (crtl). Cells were subjected to EdU incorporationassay and PI staining prior to flow-cytometry analysis. (**A**) Representative flow cytometry profiles for A549 cells. (**B**) Quantification of EdU incorporation. Results shown are average ± SEM from three independent experiments. Statistical analysis: unpaired, two-tailed Student's *t*-test with Welch correction ($^{*}p < 0.05$, $^{**}p < 0.01$, $^{***}p < 0.005$).

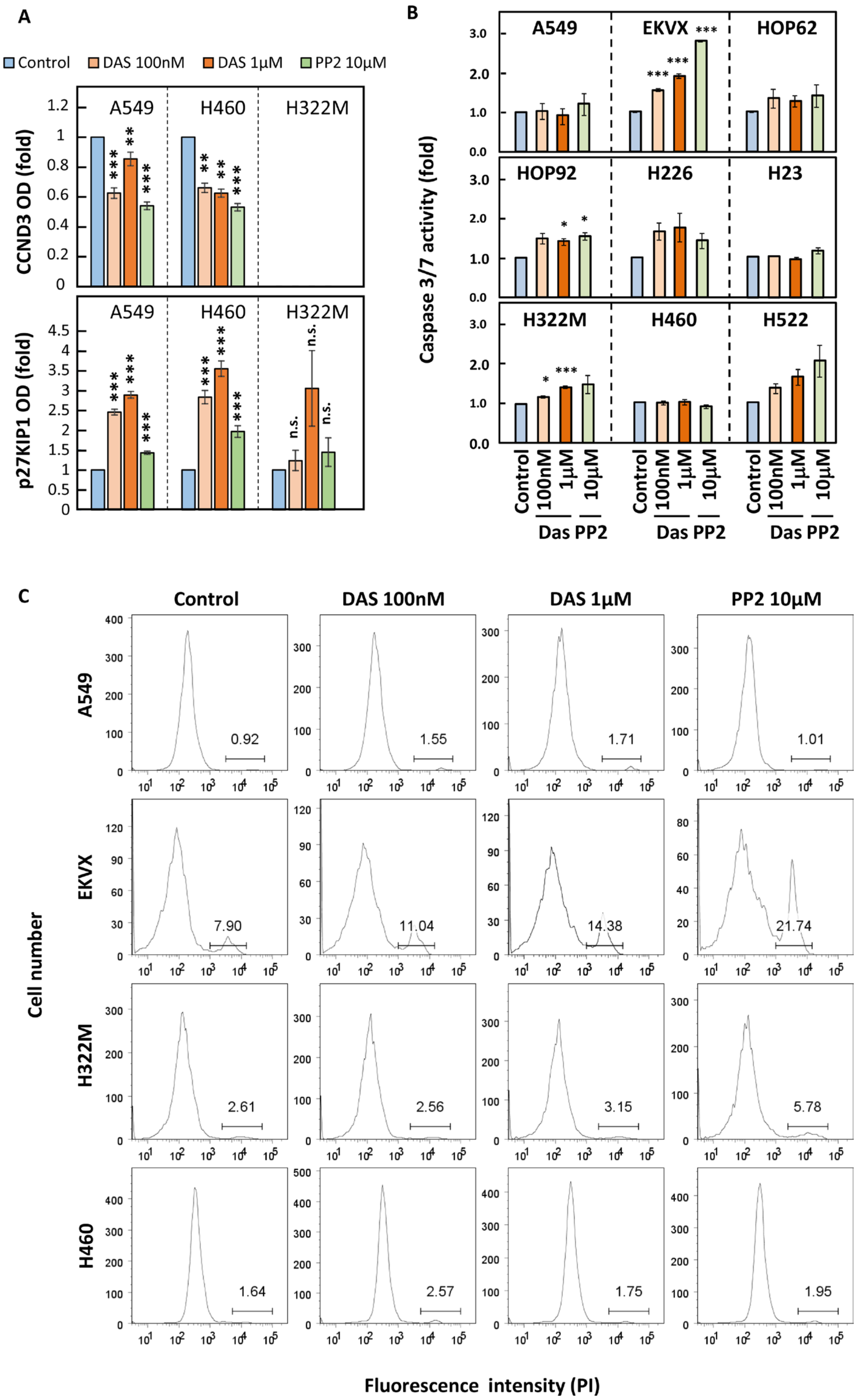

Supplementary Figure 6: Treatment with dasatinib and PP2 impacts on CCND3 and p27KIP1 expression and induces apoptosis only in some NSCLC cell lines. The indicated cell lines were treated for 24 (**A** and **B**) or 48 h (**C**) with or without dasatinib (DAS) or PP2 at the indicated concentrations. Treatment with DMSO alone was used as control (crtl). (A) Optical densitometry of triplicate blots such as those presented in Figure 4B. Data are means ± SEM. (B) Caspase 3/7 activity was assessed using a pro-luminescent caspase substrate added to cell lysates. Data are means ± SEM of three independent experiments performed in triplicate and normalised to control. (C) Cells were then incubated with propidium iodide for 15 min, harvested and analysed by flow cytometry. FACS profiles are representative of three independent experiments. Statistical analysis: unpaired, two-tailed Student's t-test with Welch correction ($^{*}p < 0.05$, $^{**}p < 0.01$, $^{***}p < 0.005$).

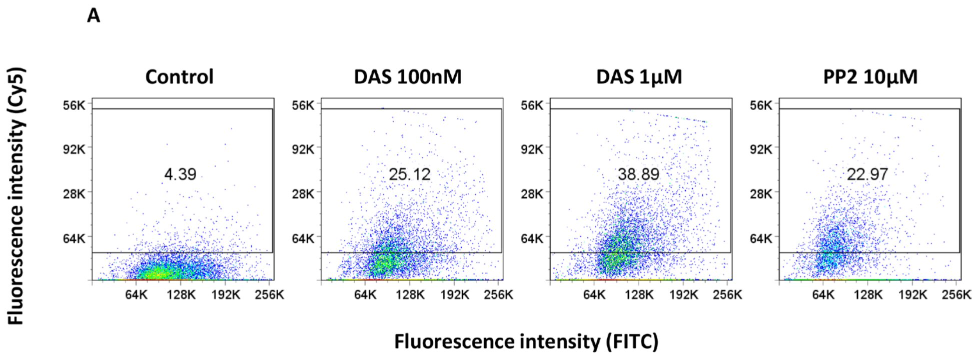

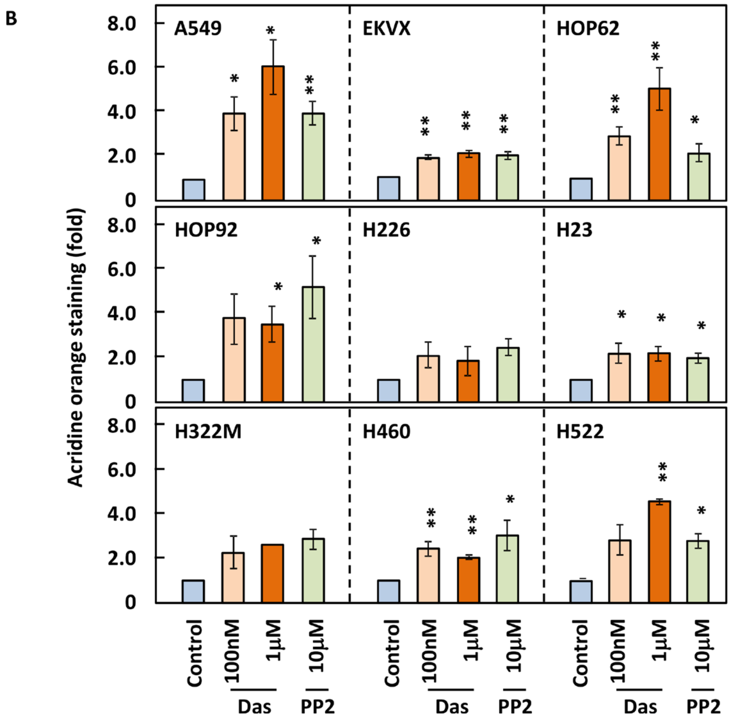

Supplementary Figure 7: SFKs inhibitors induce autophagy in NSCLC cell lines. The indicated cell lines were treated for 16 h with or without dasatinib (DAS) or PP2 at the indicated concentrations. Treatment with DMSO alone was used as control (crtl). Cells were then incubated with acridine orange for 15 min prior to harvesting. Live cells were analysed by flow cytometry for red and green fluorescence, corresponding to lysosomal acidicity and DNA content, respectively. (**A**) Representative FACS profiles from three independent experiments for A549 cells are shown. (**B**) Data are means ± SEM of red fluorescence from three independent experiments, normalised to control. Statistical analysis: unpaired, two-tailed Student's *t*-test with Welch correction ($^{*}p < 0.05$, $^{**}p < 0.01$, $^{***}p < 0.005$).

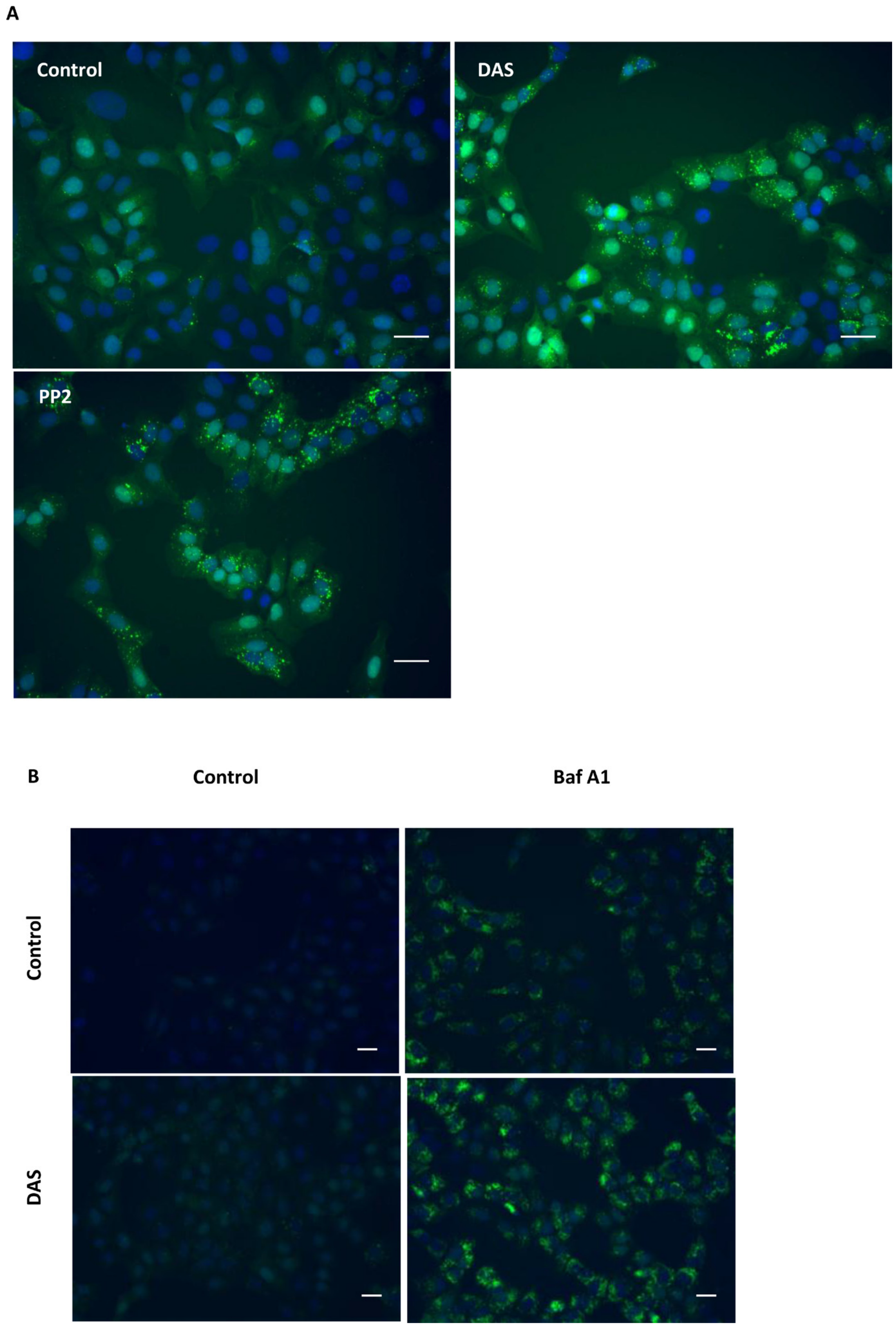

Supplementary Figure 8: Treatment with dasatinib or PP2 induces autophagy in U2OS cells. U2OS cells stably expressing LC3-GFP were incubated with 100 nM dasatinib (DAS), 3 nM bafilomycin A1 (Baf A1) or 10 µM PP2 for 16 h. Cells were fixed and stained with DAPI to visualise nuclei. Images were acquired with a ×20 objective. Blue; DAPI staining. Green; LC3-GFP labelled autophagosomes. (**A**–**B**) Representative images from 30 fields of view per condition. Scale bars; 20 µm.

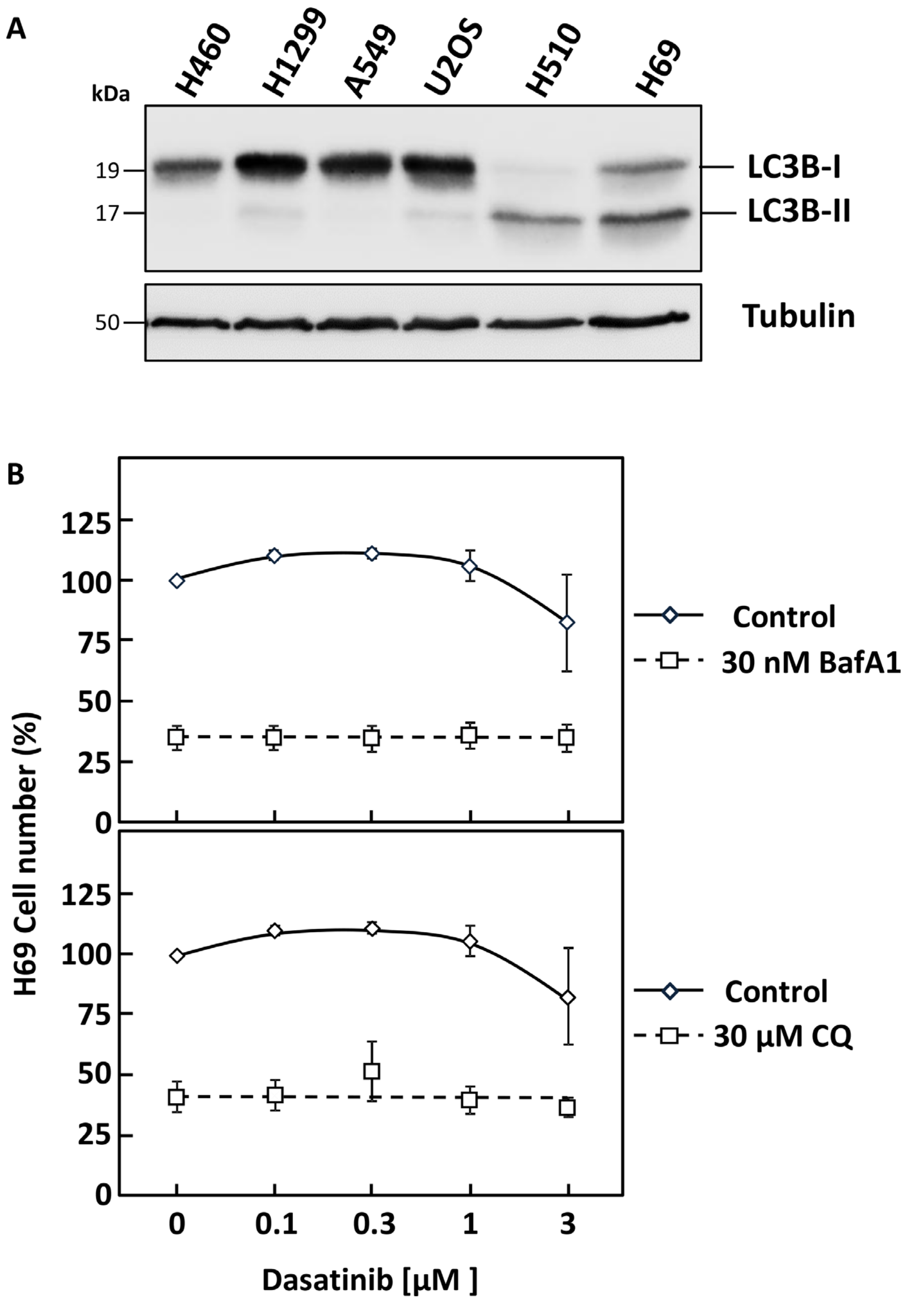

Supplementary Figure 9: Inhibition of high background autophagy in SCLC cells decrease their survival. (**A**) Whole-cell lysates from the indicated cell lines were analysed by SDS-PAGE/Western blotting for LC3. Detection of β-actin served as a loading control. (**B**) H69 cells grown in in SITA medium were pre-treated with the indicated concentrations of bafilomycin A1 or chloroquine for 2 h and then incubated or not with increasing concentrations of dasatinib for 7 days. Cell viability was measured using the WST-1 assay. Graphs represent means ± SEM from three independent experiments performed in triplicate and normalised to vehicle-only control.

A

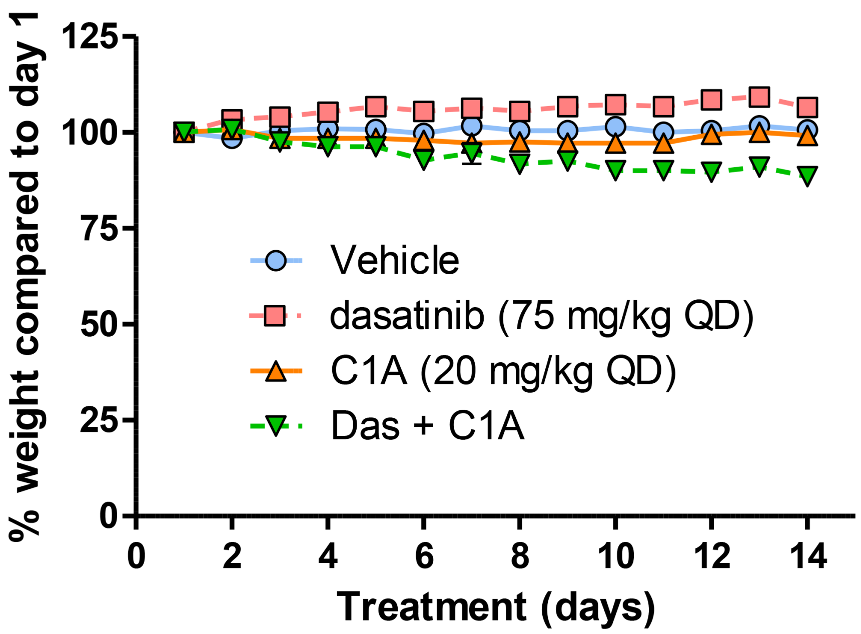

B

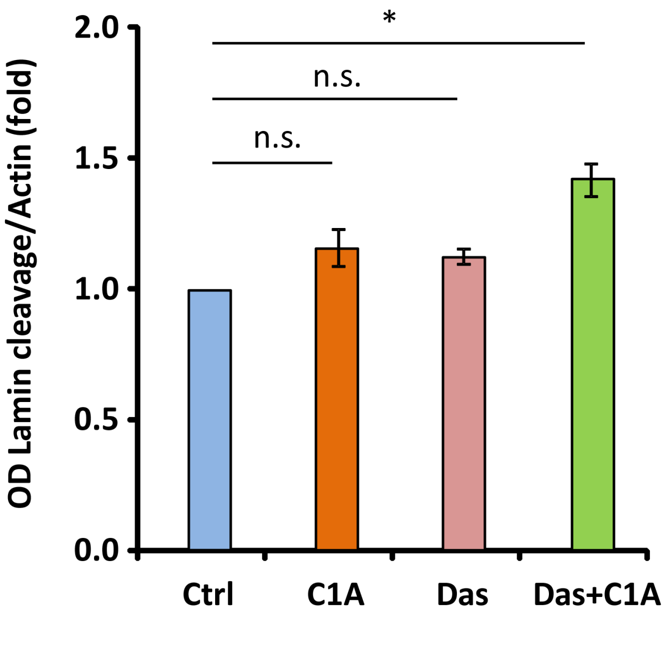

Supplementary Figure 10: (**A**) Intraperitoneal daily administration of C1A does not cause general toxicity in mice. A549 cells were injected subcutaneously in nude mice and treatment initiated when tumours reached 50–100 mm^3 with or without 20 mg/kg C1A by daily oral gavage for 2 weeks. Tumour volumes were determined by caliper measurement. $N = 5$ per condition. (**B**) Treatment with combination of C1A and Dasatinib induces intratumoural Lamin B cleavage. Lysates from tumours of treated animals where analysed by Western blotting for Lamin B and Actin. Optical densitometry was performed under ImageJ and the levels of Lamin B cleavage where normalised to those of Actin in each sample. The bar graph represents the means ± SEM of three tumour samples per condition. Statistical analysis: Student *t*-test. $^*p < 0.05$.

Supplementary Table 1: Comparison of the two databases*

	**TMA1** *N* **= 146**	**TMA2** *N* **= 138**
**Age (yrs)**	66.0 (61.3–71)	72.7 (64.9–77.7)
**Follow-up time (months)**	39.0 (18.9–76.8)	58.2 (24.4–87.6)
**Dead**	97 (66.4%)	84 (60.9%)
**Stage**		
Stage I	94 (64.4%)	71 (51.4%)
Stage II-IV	51 (34.9%)	63 (45.7%)
Missing	1 (0.7%)	4 (2.9%)
**Grade**		
Grade 1/2	52 (35.6%)	70 (50.7%)
Grade 3/4	87 (59.6%)	51 (37%)
Missing	7 (4.8%)	17 (12.3%)
**Cancer type**		
ADC	43 (29.5%)	105 (76.1%)
SCC	60 (41.1%)	33 (23.9%)
LCC	31 (21.2%)	0 (0%)
Other	12 (8.2%)	0 (0%)
**Gender**		
Male	0 (0%)	102 (73.9%)
Female	0 (0%)	36 (26.1%)
Missing	146 (100%)	0 (0%)

*Data shown are count (percentage) for categorical variables and median (inter-quartile ranges) for continuous variables.
